# A Tailored Web- and Text-Based Intervention to Increase Physical Activity for Latino Men: Protocol for a Randomized Controlled Feasibility Trial

**DOI:** 10.2196/23690

**Published:** 2021-01-29

**Authors:** Kim M Gans, Akilah Dulin, Vanessa Palomo, Tanya Benitez, Shira Dunsiger, Laura Dionne, Gregory Champion, Rachelle Edgar, Bess Marcus

**Affiliations:** 1 Department of Human Development and Family Sciences University of Connecticut Storrs, CT United States; 2 Department of Behavioral And Social Sciences Brown University School of Public Health Providence, RI United States; 3 Cardiovascular Center for Research and Innovation Tufts University Medical Center Boston, MA United States

**Keywords:** physical activity, Latino, Hispanic, men, eHealth, expert system, internet, text messaging, mobile phone, social media

## Abstract

**Background:**

Latino men in the United States report low physical activity (PA) levels and related health conditions (eg, diabetes and obesity). Engaging in regular PA can reduce the risk of chronic diseases and yield many health benefits; however, there is a paucity of interventions developed exclusively for Latino men.

**Objective:**

To address the need for culturally relevant PA interventions, this study aims to develop and evaluate Hombres Saludables, a 6-month theory-based, tailored web- and text message-based PA intervention in Spanish for Latino men. This protocol paper describes the study design, intervention, and evaluation methods for Hombres Saludables.

**Methods:**

Latino men aged 18-65 years were randomized to either the individually tailored PA internet intervention arm or the nutrition and wellness internet control arm. The PA intervention included 2 check-in phone calls; automated SMS text messages; a pedometer; a 6-month gym membership; access to a private Facebook group; and an interactive website with PA tracking, goal setting, and individually tailored PA content. The primary outcomes were feasibility, acceptability, and efficacy (minutes per week of total moderate-to-vigorous PA assessed via the ActiGraph GT3X+ accelerometer worn at the waist and 7-day physical activity recall at baseline and 6 months). Secondary outcomes examined potential moderators (eg, demographics, acculturation, and environmental variables) and mediators (eg, self-efficacy and cognitive and behavioral processes of change) of treatment effects at 6 months post randomization.

**Results:**

This study was funded in September 2016. Initial institutional review board approval was received in February 2017, and focus groups and intervention development were conducted from April 2017 to January 2018. Recruitment for the clinical trial was carried out from February 2018 to July 2019. Baseline data collection was carried out from February 2018 to October 2019, with a total of 43 participants randomized. Follow-up data were collected through April 2020. Data cleaning and analysis are ongoing.

**Conclusions:**

We developed and tested protocols for a highly accessible, culturally and linguistically relevant, theory-driven PA intervention for Latino men. Hombres Saludables used an innovative, interactive, web- and text message–based intervention for improving PA among Latino men, an underserved population at risk of low PA and related chronic disease. If the intervention demonstrates feasibility, acceptability, and preliminary efficacy, we will refine and evaluate it in a larger randomized control trial.

**Trial Registration:**

Clinicaltrials.gov: NCT03196570; https://clinicaltrials.gov/ct2/show/NCT03196570

**International Registered Report Identifier (IRRID):**

DERR1-10.2196/23690

## Introduction

### Background

Engaging in regular physical activity (PA) exerts health benefits, including decreases in all-cause mortality, obesity, and risk for other chronic diseases, such as cardiovascular disease, type 2 diabetes, certain cancers, obesity, hypertension, osteoporosis, osteoarthritis, depression, and dementia [1]. Compared with White non-Latino men, more Latino men do not meet national PA guidelines (49.5% vs 38.9%) for leisure time PA [2,3]. Although several studies show that disparities in overall PA are not as pronounced in Mexican American men when measuring PA objectively, overall levels of PA are still too low in this population [4-7]. Latino men are disproportionately burdened by PA-related health conditions, such as obesity and overweight status (81.8% vs 75.3%) [8] and type 2 diabetes (12.5% vs 7.5%) [9]. This lack of PA signifies a substantial public health problem. Furthermore, there is heterogeneity across Latino subgroups. National data have revealed that Cubans and Dominicans had the lowest leisure time PA levels, whereas Mexican Americans were the most active [10]. Thus, Latino men from subgroups other than Mexican Americans may be at even higher risk for inactivity.

Although several studies have demonstrated the efficacy of culturally and linguistically appropriate, individually tailored PA interventions for Latina women [11-15], PA intervention studies with Latinos have excluded male participants or had limited numbers of men [16-18]. Most PA interventions with Latinos feature activities perceived by participants as more traditionally feminine (eg, dance classes) and targeted more female-specific barriers (eg, childcare duties) [19]. Multiple systematic reviews have found that no PA interventions have specifically targeted Latino men [16-18,20]. Since the last review published in 2019 [18], 2 small PA interventions (n=45-50) with mostly Mexican American men have been published. One study included an intensive in-person intervention, which has limited scalability [21], whereas the other involved individually tailored print materials and text messages sent to participants on a tapered schedule for 6 months [22].

This study addresses the scarcity of interventions designed to increase PA specifically for diverse Latino men and uses technology to improve reach and accessibility. Computer-based, expert system–driven, theory-based interventions use participant-supplied data to generate messages tailored to the individual needs of each participant [23]. They have shown great promise for providing effective, widely available, and low-cost health promotion programs [24,25]. This approach may appeal to Latino men as it addresses barriers identified in formative research, such as lack of time, family involvement, work responsibilities, and transportation [26-28], using a technology-based (internet and cell phone) tailored intervention. Technology-based approaches can help overcome PA barriers reported by Latinos in our formative research (eg, lack of time and transportation) and may be especially appropriate given the rapid rise in recent years in internet use among this population. In fact, as of 2019, a large majority of Latinos reported using the internet (86%) [29] and owning a cell phone (96%) [30]; smartphones accounted for 79% of cell phones [30]. Latinos are also more likely than non-Latino Whites to use their mobile devices (smartphones or tablets) to access health information [31], suggesting that technology-based PA interventions may be especially appealing to Latino men.

Recent meta-analyses (and a comprehensive review) have described the impact of web-based interventions on PA and have found small to moderate positive effect sizes [32-34]. For mobile device–based PA interventions, one meta-analysis found a moderate effect size (g=0.54) [35]. In addition, a systematic review of texting interventions found that strong evidence exists for integrating text messages into PA trials [36]. Thus, an individually tailored, multimedia web- and text-based intervention has the potential to broadly reach Latino men at a relatively low cost, which could help reduce low PA–related health disparities.

### Objectives

The purpose of this protocol paper is to describe the study design, intervention, and evaluation methods for Hombres Saludables, an internet- and text-based tailored Spanish language intervention designed for an ethnically diverse population of Latino men (ie, Caribbean and Central and South American origin) to increase total PA. This intervention was adapted from our culturally and linguistically appropriate, internet-based PA intervention for Latina women, Pasos Hacia la Salud [14], which successfully increased and maintained total PA levels in Latina women over 12 months [15,37].

## Methods

### Overall Design

Hombres Saludables is a 6-month randomized controlled trial (RCT) for Latino men comparing an individually tailored, internet- and text-based PA intervention with a control group that received an attention-matched intervention about nutrition and wellness. The primary aims of the study are to determine the feasibility, acceptability, and preliminary efficacy of the intervention, as well as the recruitment, implementation, and evaluation protocols. Secondary aims include examining potential moderators (eg, demographics, acculturation, and environmental variables such as the neighborhood and socioeconomic environments) and mediators (eg, self-efficacy and cognitive and behavioral processes of change) of treatment effects at 6 months post randomization. The targeted sample size for this pilot trial is 50 Latino men.

### Design Considerations

To inform the design of this study, we conducted 8 focus groups with 38 Latino men in Rhode Island. We asked their opinions on potential design elements of this study, including website functionality, use of text messages, email and social media, and content for both the intervention and control arms of the study. All focus groups were audio-recorded, and the recordings were transcribed, translated, and subjected to several stages of analytic coding using ethnographic methods by 2 graduate students. Transcripts were initially read as texts to isolate obvious themes and then subjected to open coding to identify additional themes and actions that are relevant for further analysis. Next, transcripts were subjected to focused coding using the subdomains identified in the earlier stages. Following coding, we worked with the coded data set and the texts to create a componential analysis that identified patterns and themes. Focus group findings were discussed with the research team and used to adapt and refine recruitment and intervention materials to be culturally appropriate for the diverse target audience. Focus group results will be discussed in another paper; however, briefly, participants expressed interest in the use of text messages rather than email to deliver information and reminders during the intervention. Focus group participants also recommended the use of a private Facebook group as a forum to post intervention content for study participants and allow them to comment and post among themselves. Focus group participants expressed strong interest in a gym membership, stating that it would help with barriers of cost and motivation to exercise.

### Participants and Eligibility

Inclusion criteria were as follows: (1) self-identification as Hispanic or Latino; (2) self-identification as male; (3) age between 18 and 65 years; (4) self-reported 60 min or less of total MVPA a week; (5) had an adequate literacy level to read study materials in Spanish, that is, scored more than 16 on the Spanish language version of the Short Test of Functional Health Literacy in Adults (S-TOFHLA) [38-40]; and (6) owned a cell phone with texting capabilities and had regular internet access via a smartphone, tablet, or computer. Eligible participants also had to agree to be assigned to either of the 2 treatment conditions.

Exclusion criteria were as follows: (1) history of myocardial infarction or angina, insulin-dependent diabetes, or hospitalization for diabetes in the past year; (2) stroke, osteoarthritis, osteoporosis, orthopedic problems, or exercise-induced asthma; (3) any other medical condition that would make MVPA unsafe; (4) hospitalization because of a psychiatric disorder in the past 3 years; (5) BMI >45; (6) planned surgery or hospitalization in the next 6 months; and (7) intake of medication that may impair PA tolerance or performance. Any questions about medical eligibility were sent to the study physician to determine eligibility. Participants who reported another family member already enrolled in another PA study being conducted concurrently by our research team were yoked during randomization to the same study arm to prevent the possibility of cross-treatment contamination.

### Theoretical Framework for the Tailored PA Internet Intervention

**Figure 1:**
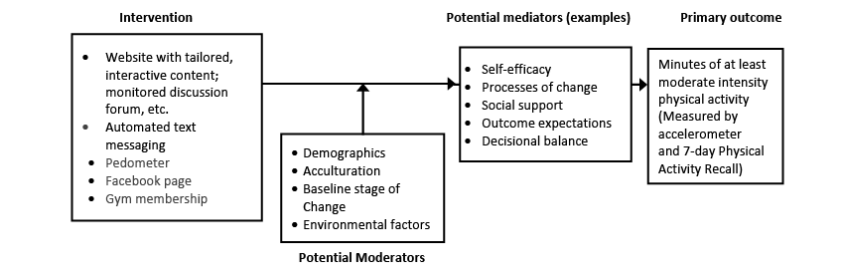
Draft intervention logic model.

The computer-based, expert system–driven, individually tailored intervention was based on social cognitive theory (SCT) [41] and the transtheoretical model (TTM) [42,43]. The intervention emphasized cognitive and behavioral strategies for increasing activity levels (eg, goal setting, increasing self-efficacy, self-monitoring, problem-solving barriers, increasing social support, and rewarding oneself for meeting PA goals). Table 1 illustrates the theoretical constructs targeted by intervention activities. In addition, the intervention logic model is shown in Figure 1.

**Table 1:** Theoretical constructs of social cognitive theory and the transtheoretical model targeted by intervention components.

Construct (theory)	Intervention component
Self-regulation (SCT^a^)	Website goal setting and PA^b^ tracking feature allow participants to log their weekly PA goals and daily activity, including minutes of MVPA^c^, and to view a graph of how their actual PA compares with their goals. Participants receive a pedometer to track their daily step count
Outcome expectations (SCT)	Web-based daily and weekly exercise tips provide information about the benefits of PA. Text messages about PA benefits
Stages of change and processes of change, for example, consciousness raising, social supports, and reinforcement management (TTM^d^)	Participants complete monthly web-based questionnaires (stages of change and processes of change) and then receive computer-based, expert system–driven, individually tailored reports for increasing their PA based on their responses
Self-efficacy (SCT and TTM)	Participants complete monthly web-based questionnaires and then receive computer-based, expert system–driven, individually tailored reports
Observational learning (SCT)	Exercise videos in Spanish (led by diverse Latino men) let men observe peers leading exercises and allow practice, which leads to an increase in self efficacy
Behavioral capability (SCT)	Text reminders to access websites, log activity, and set goals; knowledge and skills information in concise tips, detailed tip sheets, text messages, and Facebook posts. Exercise videos in Spanish (led by diverse Latino men) teach skills that help participants learn to be more physically active.
Reciprocal determinism (SCT)	Information provided about the built environment: list on website of places to be active near participants’ home; Facebook messages about community PA events
Outcome expectations and self-efficacy and perceived barriers (SCT)	Share motivational and culturally relevant information about benefits and how to address barriers in concise tips, detailed tip sheets, text messages, and Facebook posts. Exercise videos to promote self-efficacy.
Social support (SCT)	Provide social interaction and support through the online community discussion forum or on Facebook where participants can write messages and interact with each other. Concise tips, detailed tip sheets, text messages, and Facebook encourage exercising with family, spouse, friends, and coworkers

^a^SCT: social cognitive theory.

^b^PA: physical activity.

^c^MVPA: moderate-to-vigorous physical activity.

^d^TTM: transtheoretical model.

### Cultural Adaptations

Study materials were culturally adapted for a diverse population of Latino men. We enhanced cultural appropriateness through the following strategies using the frameworks described by Kreuter et al [44] and Resnicow et al [45]:

1. Peripheral or structural: We include appropriate physical activities, illustrations, role models, etc that are targeted for Latino men.
2. Evidential: We enhance perceived relevance by presenting evidence of the impact of a sedentary lifestyle for Latino men.
3. Constituent involving: We employ project staff who are Latino, including 1 Latino male research assistant.
4. Linguistic: We translate all study materials into Spanish that is appropriate for the Latino subgroups (eg, Dominican and Puerto Rican) in New England.
5. Sociocultural or deep structure: We incorporate cultural values and beliefs to provide context and meaning (eg, content about gender role expectations, conflicts with family time, and partner support) [12,44-53].

In designing intervention materials (eg, the web-based interface), we integrated components of the cultural dimensions theory by Hofstede, a framework for cross-cultural communication that shows the effects of the culture of a society on the values of its members and how these values relate to behavior [54]. In addition, our intervention addressed substantial PA barriers reported by Latinos in our formative research (ie, stress reduction, work and family time conflicts, lack of time, and accountability). Although similar PA barriers have been reported in non-Latino populations, in both men and women [55-58], adaptation of intervention content for cultural and linguistic relevance to Latino men was still required. Table 2 provides a description of how the intervention materials were culturally tailored.

**Table 2:** Cultural adaptations for the Hombres Saludables physical activity intervention.

Theme		Intervention modification
**Surface structure**		
	Activity preferences	Focus intervention on gender-neutral and male-associated activities (eg, soccer and hiking)
	Language	Translate intervention into appropriate Spanish for the diverse Latino audience
	Literacy	Use qualitative methods (eg, focus groups with Latino men to review intervention materials) and low-literacy strategies (eg, Flesch Kincaid grade level less than eighth grade) to modify measures and materials to better match educational experience of participants
	Role models	Provide videos, photos, and stories of diverse Latino men and families from the target population on a website
**Deep structure**		
	Gender role expectations	Emphasize the need for the head of family to set a good example by being active and protect family members by exercising with them; highlight benefits of aerobic activities for men’s health
	Not wanting to spend money on fitness when that money should be used for family needs	Reframe PA^a^ to include behaviors that do not require gym membership or special equipment, distribute information on low- or no-cost PA resources in the community (eg, hiking and walking trails, recreation centers, and pickup soccer games), offer a list of free Spanish or bilingual smartphone apps that provide access to exercise resources, and provide a 6-month gym membership
	Perceived lack of access to culturally appropriate PA	Provide tailored community guides identifying places to do PA, including information and schedules for free and low-cost team sports at local recreation centers and community or social sports leagues; include Spanish exercise videos that are appropriate for Latino men; offer a list of free Spanish or bilingual smartphone apps that provide access to exercise resources; and provide a 6-month gym membership
**Barriers to PA**		
	Stress reduction	Provide information on PA and stress reduction for stressors commonly experienced by Latino men
	PA conflicts with work schedule	Provide tips for exercising at work or for transportation and on finding time with a hectic schedule; highlight low-cost local sports and activities that occur on nights and weekends
	Lack of time and conflicts with family time	Augment existing content on this topic with examples that are familiar to Latino men (planning PA around family and work commitments); share Spanish exercise videos that are appropriate for Latino men; and provide membership to a gym that is open for extended hours, which helps men find time to exercise around work schedules. Provide tips and texts for getting children and family involved in PA and include specific suggestions regarding family-friendly activities (eg, easy hiking trails)
	Partner support	Provide tips on eliciting social support from friends and family
	Need for accountability	Provide pedometer, emphasize monthly personal reporting and feedback based on participant’s reported PA (steps or minutes), provide normative feedback comparing their progress with others, send regular text messages asking about recent activity and reminding participants to log activity and answer monthly surveys, and offer private Facebook group to post progress and request support

^a^PA: physical activity.

### Tailored PA Internet Intervention Arm

#### Website

Participants in the tailored PA internet intervention group received access to the Hombres Saludables study website. All website content was published in Spanish and adapted to be culturally and linguistically relevant for Latino men. The website was developed to be mobile phone friendly. Participants in this study arm were asked to log their minutes of MVPA each day. They were also asked to set a weekly PA goal and log it on the website. The website offered a goal setting and tracking feature to allow participants to view graphs of their actual level of PA compared with the goals they set each week. Participants were asked to complete monthly questionnaires on the study website, assessing key theoretical components of SCT and the TTM. Answers from these questions generated their individually tailored PA reports. These reports were published on the website automatically upon completion of each monthly survey. The reports used a bank of more than 300 messages from the computer-based, expert system and offered feedback on (1) the current stage of motivational readiness for PA, (2) self-efficacy, and (3) cognitive and behavioral strategies associated with PA.

The computer-based, expert system also provided feedback on how the participant compared with individuals who are physically active based on American College of Sports Medicine guidelines [1] of engaging in the equivalent of 150 min a week of MVPA (normative feedback) and how the participant compared with their earlier responses (progress feedback) [14]. A few days after receiving the tailored report, a web-based motivation-matched PA manual based on the TTM stage of change for each participant was published on their individual website account. The manual emphasized cognitive and behavioral strategies for increasing activity levels such as goal setting, self-monitoring, problem-solving barriers, increasing social support, and rewarding oneself for meeting PA goals (eg, nonfood rewards) [14]. Although the ultimate goal of the intervention was increasing total PA, content focused mostly on increasing leisure time–, lifestyle-, and transportation-related PA rather than occupational PA.

Other website features included resources to promote PA. Participants could access city guides containing useful information on where to be active in their city (eg, parks, bike paths, gyms, and local recreation centers), a list of free PA promotion apps participants could download on their smartphones, a series of Spanish language exercise videos found on YouTube, and concise daily and weekly tips published on the website throughout the 6-month intervention as well as detailed 2- to 3-page *tip sheets*. In the first 2 months, all participants received the following tip sheets: how to set achievable goals for PA, finding the time for PA, how to fit in short sets of PA throughout the day, and motivating yourself to be more physically active. Then, during their first monthly survey on the website, participants chose to receive up to 15 tip sheets of interest selected from a list of 20. Those tip sheets were then published on their website account throughout the intervention on a weekly or biweekly basis. Sample topics that participants could choose to receive included stretching, being active as a family, tips to exercise correctly, exercising outside, rewarding yourself for achieving your PA goals, etc. In addition, the website contained a community forum (for participants to write messages and interact with each other), an *Ask the Expert* area (where participants could ask a question and study staff post responses), and a section where participants could alert the staff if they sustained an injury during the study. Participants were also given a pedometer to help track their daily steps; alternatively, participants could choose to log daily steps using an app on their smartphone. See Multimedia Appendix 1 for screenshots of the website.

#### Text Messages

Throughout the 6 months of the intervention, participants received text messages 4 to 6 times per week. These included prompts to access new information posted on the website, such as their tailored tip sheets; reminders to log their minutes, set a weekly goal, and complete their monthly questionnaire; and texts that were informational and motivational, for example, suggestions to overcome barriers and enlist social support. Examples of the latter included: “Is lack of time a problem for you? Try waking up 15 minutes earlier to take a short 15-minute walk, and then another 15 minutes at lunchtime or in the afternoon for a second walk;” “There are many exercise videos on YouTube and in mobile apps. Look in the ‘Ways to Be Active’ section on the website for our recommendations for exercise videos and apps;” and “Remember that physical activity burns calories, improves sleep, increases your energy, and reduces stress.”

#### Facebook Group

Participants were also given the option to join a private Facebook group where study staff posted weekly to the group with tips, quizzes, events, and information related to PA. Participants were encouraged to engage with the content, submit questions, and post comments. Each month, a random participant who had engaged with the Facebook content won a US $25 incentive.

#### Gym Membership

Participants also received a voucher for a free 6-month membership worth US $60 to a local gym franchise with multiple locations. Participants who did not live in the surrounding Rhode Island or Massachusetts area received US $60 toward the costs of a gym membership.

#### Check-In Phone Calls

One week after enrolling in this study, intervention participants received a phone call from the study staff. The 5 to 10 min call ensured that all aspects of the website, Facebook, and text message alerts were functioning well, and the study staff answered any participant questions. After 1 month, the participants completed another 10 to 15 min call with study staff to review their progress and answer any study questions. A staff member supported the participants in creating a new goal for the second month of the study. If any barriers to PA arose, the staff member assisted the participants in developing solutions.

### Attention-Matched Nutrition and Wellness Internet Group (Control Group)

**Figure 2:**
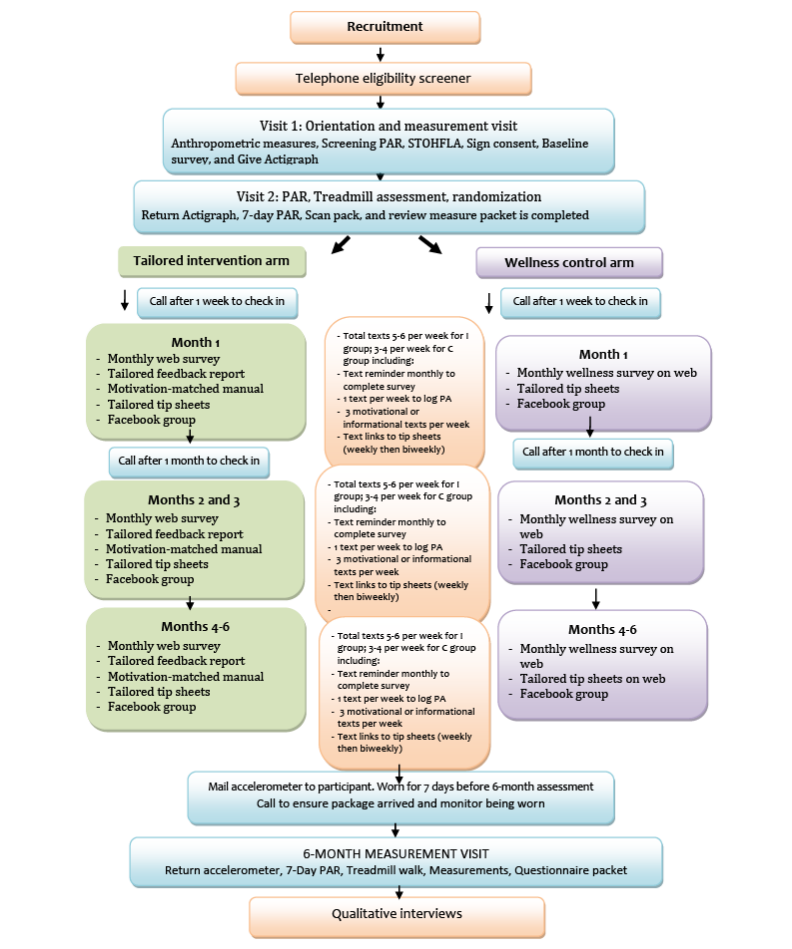
Hombres Saludables study flow diagram. C: control group; I: intervention group; PAR: physical activity recall; STOHFLA: Short Test of Functional Health Literacy in Adults.

Participants randomized into the control arm received access to a Spanish language website with information on nutrition and men’s health and wellness topics unrelated to PA. The study staff guided participants through the website and set up their account. The website featured healthy recipes; app suggestions for healthy eating; and weekly tips on topics such as eating more fruits and vegetables, healthy drink choices, reducing salt intake, sleep health, prostate care, information about cholesterol, and supplements. Tip sheets on similar topics were also regularly posted on the website throughout the intervention on the same schedule as the intervention arm. Participants were asked to complete a monthly survey on the website on the same schedule as the PA arm. Survey questions asked about diet, sleep, and wellness habits. Participants completed the first survey during the baseline visit. Control arm participants also received text message alerts 3 to 4 times per week with reminders of new information on the website and helpful tips. Participants also received access to a private Facebook group that offered additional information on nutrition- and wellness-related topics. This group also received a check-in call from the study staff 1 week and 1 month after enrollment (Figure 2).

### Feasibility Trial

#### Participant Recruitment

We used various recruitment strategies in Rhode Island, Connecticut, and Massachusetts as well as nationally. Locally in Rhode Island, Connecticut, and Massachusetts, we advertised on Craigslist and posted flyers in local community organizations and businesses (eg, restaurants, barbershops, grocery stores, and laundromats). We worked extensively with the local library systems and presented at local high school equivalency certificate, citizenship, and English language classes. We also attended local church groups, elementary schools, Latino men’s groups, and community organizations working with the Latino population. We visited worksites of companies with high Latino employment and posted information in college campus student centers and on student listservs at various local colleges. We also ran paid radio ads on a local Spanish language FM radio station, performed a guest stint on a Latino radio talk show, and left study flyers and brochures with doctors’ offices and health clinics. Due to a slower recruitment rate than anticipated, we also expanded recruitment efforts nationally by publishing paid ads on Facebook and Craigslist pages for different towns and cities, mainly focusing on the East Coast. Use of web-based recruitment, particularly the use of Facebook, has been shown to be an effective approach for recruiting participants in health research [59-61].

#### Visit 1

A summary of the study and flow diagram is shown in Figure 2. Interested participants were screened over the phone for eligibility. Once eligibility was determined, potential participants were scheduled for their first visit. Local participants attended visits in person. Participants who lived too far away to attend in-person visits, hereafter referred to as *distance participants*, completed this visit by phone. We offered flexible scheduling for visits during weekends and weekdays and nights, both in person and via phone.

For participants attending visit 1 in person, the bilingual and bicultural research staff gave an overview of this study, described study steps and rights of participation, and answered participant questions. The participant then underwent measures to further assess eligibility, including height, weight, and waist circumference measurements; the S-TOFHLA literacy questionnaire; a brief 7-day physical activity recall (PAR) [62,63] listing the minutes of total MVPA they had done for each day of the previous week to assess their current activity level; and a basic text and web accessibility check to ensure that they were capable of using the internet and receiving text messages. A participant who did not score higher than 16 on S-TOFHLA or whose measurements calculated a BMI at 45 or higher become ineligible during this visit. Participants were also ineligible if they reported more than 60 weekly min of total MVPA. Eligible participants then signed the informed consent document. After this, they completed the baseline survey including demographic characteristics and questionnaires on PA-related psychosocial variables (stages and processes of change, self-efficacy, enjoyment, social support, stress, neighborhood cohesion, police profiling, and neighborhood safety). At the end of the visit, eligible participants received an ActiGraph wGT3X-BT accelerometer, with instructions to wear the accelerometer on their waist with a Velcro belt during waking hours for 7 consecutive days. Participants were also given a form to write down the dates and times they put on and took off the accelerometer. At the end of the visit, participants received a Clincard, a reloadable prepaid card (similar to a debit card) for monetary incentives, and a sheet of frequently asked questions and answers about the study. We then scheduled their visit 2 to occur approximately 8 days later.

For visit 1, *distance participants* were mailed a copy of the informed consent document and a hard copy of S-TOFHLA to their home, ahead of their scheduled phone call. The study staff reviewed the informed consent document and then received verbal consent from the participants. Study staff asked these participants to self-report their height and weight. Waist circumference measurements were not collected from distance participants. The participants completed the S-TOFHLA while on the phone with the study staff. After the phone call, the hardcopy document was mailed back to the study office. The staff member then administered the baseline survey questionnaire over the phone. For participants who had a personal computer, we offered the option to email a link for the participant to complete the survey themselves. After the phone call, eligible *distance participants* were mailed the accelerometer and a log form, as mentioned above, with instructions to wear the device for 7 consecutive days and a return envelope to mail the device and log form back immediately afterward.

#### Visit 2 (PA Assessment and Randomization Session)

In-person participants returned for a second visit approximately 8 days after the first visit. They brought the accelerometer and the wear-time log form to the visit. Any participant with insufficient wear time (<3000 min over 4 days or <5 days of 600 min each) was asked to rewear the accelerometer, and the visit was rescheduled. Participants with sufficient wear time completed a 10-min treadmill walk to demonstrate moderate-intensity PA (3-4 miles per hour). Heart rate and rate of perceived exertion were documented throughout the treadmill walk by the study staff. The goal of the walk was to help improve the accuracy of participants’ self-report of their PA by providing a real-time demonstration of a 10-min bout of moderate-intensity PA with no breaks. The protocol for this demonstration was developed by Dr Marcus and has been used in earlier studies [11-13,64]. Participants then completed a 7-day PAR [62,63]. If participants reported more than 60 min of total MVPA in bouts lasting 10 min or more, they became ineligible.

Participants were then randomized to 1 of the 2 Spanish language internet and text message–based conditions: tailored PA intervention arm or nutrition and wellness control arm. Group assignment was determined using a permuted block randomization procedure with small randomly sized blocks. Randomization was stratified by the TTM stage of change [42,43] to ensure an equal distribution of treatment assigned across levels of motivational readiness for PA.

At the end of visit 2, the study staff helped participants in the tailored PA intervention arm to set up their personalized website account. The study staff also set up a bookmark to the page on the participant’s smartphone to aid easy access to the website and then provided thorough instructions on using all sections of the website. In the final phase of the visit, the study staff helped the participants set a personalized exercise goal and create a detailed PA plan for their first week. The staff members and the participants discussed potential barriers to completing this goal and how to overcome those barriers. The staff members walked the participants through these goal-setting steps and how to record their minutes of MVPA and goal on the website to ensure that the participants were able to complete these steps independently throughout the 6-month intervention.

At the end of the visit, the staff members reviewed the study goals and expectations and asked the participants to do the following:

1. Try to do MVPA for at least 10 min at a time, with no breaks.
2. Track how much exercise they perform each day and log the time on the website.
3. Wear the pedometer every day and log the steps on the website.
4. Review and revise the exercise goal each week to work up to 150 min of MVPA each week by the end of 6 months in the study.
5. Complete the monthly questionnaire on the website.

*Distance participants* completed visit 2 by phone and were guided through the same steps by the study staff. The visit was broken into 2 parts. Part 1 was completed on the day immediately after 7 consecutive days of accelerometer wearing. A staff member gave the participant a detailed explanation of what MVPA feels like, including examples of activities at this level. The staff member then completed the PAR by phone. The participant was instructed to return the accelerometer by mail, with a preaddressed envelope. Once the device was received back at the office, the study staff reviewed the data to ensure sufficient wear time and then scheduled part 2 of the visit by phone. In part 2, the *distance participant* was randomized into one of the study arms using the same randomization procedure as local participants, and then, the staff member guided the participant through the website and other study components by phone.

#### Study Incentives

For participants traveling to our office for in-person visits, we offered a US $10 incentive to aid with the cost of transportation and a monthly US $5 incentive to aid with cell phone and data costs. Participants were also compensated for their time at evaluation time points, receiving US $25 for completing their second visit and US $50 for completing the 6-month assessment visit. In addition, participants received US $10 for returning their accelerometer at visit 2 and at the 6-month visit. Each time the participants completed a monthly web-based questionnaire, they also received a US $10 incentive. Local participants received a voucher for a free 6-month gym membership worth US $60, and distance participants received US $60 toward the cost of a gym membership. Those in the PA intervention arm received the gym membership at the start of the study, whereas those in the control arm received the membership (or financial equivalent) at the 6-month follow-up.

#### 6-Month Visit

At the end of the 6-month intervention, participants were contacted again to set up their final assessment visit. Participants were mailed an ActiGraph accelerometer and asked to wear it for 7 complete days, following the baseline protocol. Participants were scheduled for their assessment visit on day 8 after they started wearing their monitor. *Distance participants* mailed the device back to our office on day 8 and conducted the visit with the study staff by phone.

At the start of the visit, the study staff reviewed the ActiGraph wear data to ensure that it was worn for sufficient time. Any participant with insufficient wear time was asked to rewear the accelerometer, and the visit was rescheduled. Participants then completed the same survey measures from the baseline assessment, with some additional process evaluation questions. Height, weight, and waist circumference were recorded again, followed by a 10-min treadmill walk (for in-person participants) and the PAR assessment. Finally, after the 6-month visit, the study staff conducted brief semistructured qualitative interviews with study participants who agreed to complete this interview.

### Measures and Outcomes

#### Demographics

Demographic questions at baseline assessed age, education, race, ethnicity, income, employment status, marital status, household size, country of birth, Hispanic subgroup, and years lived in the United States. In addition,, the Brief Acculturation Scale [65] asked 4 questions about languages used in different contexts.

#### PA Outcomes

The primary outcome measure is total PA, as measured by an accelerometer (ActiGraph wGT3X-BT). All participants were asked to wear an accelerometer for 7 days to measure their movement and intensity of activity. The minimum acceptable wear time is 5 days, with at least 600 min daily, or 4 days, with at least 3000 min total. The daily and weekly minutes of MVPA were calculated with Actilife software, using a minimum cutoff point of 1952 [66] to define the moderate-intensity PA and a minimum activity bout of 10 min, as current recommendations suggest that MVPA activities should last at least 10 min at a time [67,68]. Accelerometers have been validated with both total energy expenditure [69] and heart rate telemetry [70].

A self-reported measure of total weekly PA was also included as an outcome measure of MVPA. Using the 7-day PAR [62,63], an interviewer asked participants about moderate, hard, and very hard activities that they might have engaged in during each morning, afternoon, and evening over the past week. The 7-day PAR has repeatedly shown acceptable internal consistency, reliability, and concurrent validity with objective measures of PA [71-75], along with sensitivity to changes [72,73] in both moderate and intensive levels of PA [74,75]. In addition, the 7-day PAR demonstrated test-retest reliability among Latino participants [76]. The 7-day PAR data were assessed to overlap with accelerometer wear to corroborate the self-reported data.

#### Psychosocial Variables

Readiness to change, self-efficacy, and processes of change were also assessed as psychosocial constructs related to PA. The 5-item PA stages of change questionnaire determines whether a participant is in the precontemplation, contemplation, preparation, action, or maintenance stage of PA change. This measure has demonstrated reliability and concurrent validity with measures of self-efficacy and current activity levels [77,78]. The 40-item processes of PA change measure asks participants how often (never, seldom, occasionally, often, or repeatedly) they engage in various cognitive and behavioral strategies associated with behavior change [79]. The measure contains 5 behavioral subscales (counterconditioning, helping relationships, reinforcement management, self-liberation, and stimulus control) and 5 cognitive subscales (consciousness raising, dramatic relief, environmental re-evaluation, self–re-evaluation, and social liberation). A 5-item self-efficacy measure was included to assess confidence in one’s ability to exercise in various situations on a 5-point scale, ranging from not at all confident to extremely confident [77]. In addition to being administered at baseline and at 6-month follow-up, the readiness to change, self-efficacy, and processes of change measures were administered on a monthly basis via the website to help generate the computer-based, expert system feedback reports for the intervention group.

Additional psychosocial measures related to PA included social support, PA enjoyment, perceived stress, and perceived quality of life. The social support measure (social support for exercise) [80] included 2 sets of 14 items (1 set for friends and 1 set for family) related to the frequency with which friends or family members provided social support for PA over the past 3 months, with response options including none, rarely, a few times, often, very often, or does not apply. The Physical Activity Enjoyment Scale [81] measures the level of enjoyment that a person derives from engaging in PA. Using a scale of 1 to 7, participants were asked to rate their feelings on 18 items about their enjoyment of PA (eg, a rating of 1 means *I find it pleasurable* and a rating of 7 means *I find it unpleasurable*). The Perceived Stress Scale [82,83] is a widely used, validated instrument composed of 10 items to measure perceived stress in the past month, with a 5-point scale, where 0 means never and 4 means very often. The perceived quality of life was measured using a single item modified from the 26-item World Health Organization Quality of Life measure [84]. Participants were asked to rate their quality of life as excellent, very good, good, or poor.

#### Neighborhood Measures

Measures of neighborhood safety, neighborhood social cohesion, neighborhood police attitudes, and fear of police were also administered. Neighborhood safety was assessed with a single item [85] asking participants if they felt safe in their neighborhood all of the time, most of the time, some of the time, or none of the time. The Neighborhood Social Cohesion [86] scale is a reliable, validated set of 4 items asking participants to rate their level of agreement with statements about their neighborhood. Neighborhood police attitudes were assessed with 7 items asking whether police activities in the neighborhood (eg, stopping too many people on the street without a good reason, stopping people because of the color of their skin, being rude to people they stop, and disrespecting women when they stop them) were a big problem, some problem, or no problem [87,88]. Fear of police was assessed with 2 questions that asked if the participant agrees or disagrees (strongly agree, agree, neither agree nor disagree, disagree, and strongly disagree) with the following statements [87]: Are you sometimes afraid that police will stop you and threaten to arrest you when you are completely innocent? and Are you sometimes afraid that police will stop and threaten to arrest one of your children, or a younger member of your family, when they are completely innocent? On the basis of our earlier work [89], a new neighborhood police attitude question developed by our team was included to ask how worried a participant was that police would stop them if they were exercising in their neighborhood. Response options to this question included not at all worried, somewhat worried, and very worried.

#### Built Environment Measures

To assess built environment factors that influence PA, including land use characteristics, sidewalks, shoulders and bike lanes, street characteristics, and quality of the pedestrian environment, we used Objective Neighborhood Audits using Google Street View [90-92] and the Active Neighborhood Checklist (ANC) [93]. We used a 0.5-mile buffer around participants’ homes. We used Google Street View to conduct the ANC audit, which has demonstrated excellent reliability with in-person audits and audits comparing new, archived, and commercial imagery [91,94].

#### Control Group Measures

For the nutrition and wellness control arm, a wellness questionnaire assessed knowledge regarding the men’s wellness topics presented in the control materials. We also assessed pre and post fruit and vegetable intake using the National Cancer Institute's *Eating at America's Table All Day Screener* [95].

#### Process Evaluation

Standardized protocols were used in training staff to conduct all study visits. All PAR questionnaires were reviewed for errors before data entry. To ensure receipt of treatment, phone calls were conducted 1 week and 1 month post randomization to ensure proper use of the pedometer and self-monitoring on the website. Participants who were not completing their PA logs or their monthly web-based questionnaires were also contacted. To measure fidelity and dose of treatment implementation—and to provide insight into participant usage—the website captured the number of log-ins and views of each page or link; how much time participants spent on the website; and what participants entered on the website, that is, goals, discussion board posts, *Ask the Expert* submissions, etc; data were tracked by user selections and time stamped accordingly. Receipt of text messages was assessed by the text messaging system, and Facebook participation was measured by counting participants’ engagement such as likes, comments, and posts.

#### Feasibility

Our main feasibility measure is a participant retention rate of 80% or more. To inform a future study, we also measured time to recruit 50 participants, what proportion of recruited participants were eligible and reasons for ineligibility, the yield of various recruitment methods, and baseline process data (proportion of eligible participants who completed study visits, length of visits, time range from initial recruitment to randomization, visits attempted and completed on different days, duration of visit, refusals, and interviewer notes on surveys). Six-month visit process data included duration of the visit, the proportion of randomized participants completing the study, and those who refused or were dropped from the study along with reasons for dropping as well as participants we were unable to reach for follow-up.

#### Acceptability

In the 6-month follow-up survey, participants completed questions regarding their overall level of satisfaction with the intervention (*In general how satisfied were you with the intervention?*); their satisfaction with each component of the intervention; the degree to which these components were accessed, read, and/or used; and how helpful they were. We also conducted a poststudy qualitative interview with the study participants to explore how they found out about the study and why they joined, their perceptions about various study protocols (recruitment, visits, treadmill walk, incentives, etc) and individual components of the intervention (eg, website components, text messages, Facebook, and gym membership), and thoughts on ideas for future interventions. Qualitative interviews were digitally recorded, with the recordings sent to a professional company for transcription and translation into English. The transcribed responses to each interview question are being thematically coded by graduate students using similar methods as the focus groups described above.

#### Power Analysis

Our sample size estimates were based on results from 2 of our completed studies with accelerometer data for Latina participants [12,15]. In addition, in a small pilot [19], the mean change from baseline to 6 months in MVPA from the subsample of male participants who were given accelerometers was 76.4 min per week (SD 113.5) for the intervention versus 15.0 min (SD 22.6) for the control. Although the effect size (*d*=0.75) was large, the subsample was small (N=9) and, thus, must be considered with caution. In our recently completed intervention with Latinas [15], the mean change in total MVPA was 42.5 min per week (SD 81.8) for the intervention versus 9.0 min (SD 45.7) for the control, yielding an effect size of *d*=0.51. With 25 participants randomized to each arm, we expected to have at least 43% power to detect differences in accelerometer-measured MVPA between conditions at 6 months, assuming an effect size of *d*=0.51 and 75% power if *d*=0.75, using a two-tailed significance α of .05. The web-based program for Latinas had a slightly higher effect size; therefore, this was a conservative estimate. Although we do not expect to achieve statistically significant group differences in this pilot study, we will determine the effect size achieved between groups and use these data to estimate the sample size needs for a future RCT.

#### Planned Analyses

The primary aim of this study is to determine the feasibility, acceptability, and preliminary efficacy of the tailored PA intervention. We will consider the intervention feasible if at least 80% of the randomized participants are retained at the 6-month follow-up. The intervention will be considered acceptable if at least 80% of participants completing the 6-month follow-up respond favorably (*satisfied* or *very satisfied*) to the question *In general how satisfied were you with the intervention?* on the 6-month follow-up survey. We also asked more questions about intervention and study acceptability on the 6-month survey and the poststudy qualitative interviews described earlier. The main efficacy outcome was minutes per week of total MVPA, as measured by accelerometer data and PAR self-report, assessed at baseline and 6 months after baseline. The hypothesis is that participants in the PA intervention condition will have greater increases in minutes of total MVPA from baseline to post intervention (6 months) than participants in the control condition.

As a preliminary step, we will assess potential between-group differences in baseline characteristics (demographics and baseline PA level) using graphical methods and nonparametric and parametric tests as appropriate (eg, the Wilcoxon rank sum test for skewed data, *t* tests for normally distributed continuous data, and chi-square tests for categorical data). Any variables not balanced by randomization will be controlled for as covariates in subsequent analyses if they are correlated with the outcome (eg, minutes per week of MVPA) at a modest *P*<.10 level. We will estimate the preliminary efficacy of intervention compared with control using a generalized linear model in which we regress minutes per week of objectively measured MVPA at 6 months on the treatment assigned, baseline value of the outcome, and potential confounders (including those variables not balanced by randomization). To avoid the effects of outliers, we apply a normalizing transformation (if necessary) to the outcome before analysis. Should this transformation not adequately bring the data toward normality, we will model the median outcome (instead of the mean) using a quantile regression model.

Modeling is performed using a likelihood or quasi-likelihood–based approach and, thus, makes use of all available data (intent-to-treat sample) to produce consistent estimates of the regression parameters. Our goal is to estimate effect sizes, rather than strict statistical hypothesis tests. A similar modeling strategy will be used to estimate effects on self-reported minutes per week of MVPA.

Potential moderators will be examined using a similar analytic approach to that described earlier. For example, the total PA at 6-month follow-up (as measured by the accelerometer) will be regressed simultaneously on each moderator (eg, neighborhood PA environment profiles), as determined by latent class analysis, treatment assignment, and the interaction between the 2. If the interaction term is nonzero, we will conclude that there is evidence for a potential moderator. Models will also control for potential confounders of the association, including baseline PA and any variables unbalanced between arms. Our interest is in estimating effect sizes for conditional effects rather than strict statistical hypothesis testing.

## Results

This study was funded in September 2016. Initial institutional review board approval was received in February 2017. Focus groups and intervention development were conducted from April 2017 to January 2017. Recruitment for the clinical trial was carried out from February 2018 to July 2019. Baseline data collection was carried out from February 2018 to October 2019, with a total of 43 participants randomized. Follow-up data were collected through April 2020. Data cleaning and analysis are ongoing, and we expect study results to be published in summer 2021.

## Discussion

### Importance of the Study

PA is a critical health behavior known to promote health and prevent the onset of chronic diseases and mortality [1,96,97]. Previous research indicates that Latino men are underserved with respect to inclusion in interventions designed to increase PA [16-18]. Therefore, this study addresses these research gaps by specifically targeting diverse subgroups of underserved Latino men (ie, Caribbean and Central and South American) and engaging them in the intervention design [10,98]. This engagement is critical as (1) the majority of PA research with Latino men to date has involved mainly Mexican Americans [21,55], and these results may not generalize to other Latino men subgroups, and (2) there may be additional cultural considerations for retention of non-Mexican Latino men and their perceived acceptability of a PA intervention [10,18,98]. As such interventions remain untested with diverse Latino men, we designed this novel tailored web- and text-based PA intervention and piloted it in a feasibility RCT with these diverse groups of Latino men. If the results of the pilot study provide support for feasibility, acceptability, and preliminary efficacy, then a larger, fully powered efficacy RCT will be tested with diverse Latino men.

The Hombres Saludables study leverages theories of behavior change and low-cost, technology-based intervention delivery mechanisms that demonstrate high reach with Latinos, most of whom use the internet and own a cell phone [29,30], although future research should consider that internet usage for health-related purposes may vary by the Latino subgroup [98]. These theoretical and technological components have demonstrated efficacy in other populations [32-34], including Latinas [11-15,37]. For Latino men, however, studies documenting the efficacy and effectiveness of such low-cost interventions are lacking [16-18]. In the limited intervention research that does exist, 98% of the 48 participants in one study identified as either Mexican or Mexican American. The intervention focused on weight loss, not specifically PA, and involved weekly in-person individual sessions with a bilingual, bicultural Hispanic male lifestyle coach, which, although effective in increasing leisure time PA, raises cost, replicability, and scalability concerns [21]. Another small study with 45 Latino participants (mostly Mexican American) delivered a 6-month intervention wherein participants received a baseline counseling session and then individually tailored PA print materials and text messages. Intervention participants increased their total MVPA significantly more than the control group [22]. Thus, the findings from the Hombres Saludables study will contribute to intervention research by providing preliminary evidence of how integration of theories of health behavior change with internet and text-based intervention components may impact PA among diverse Latino men, a disparity group with low levels of leisure time PA [2,10].

With regard to the intervention dose and components of the study design, literature findings indicate that interventions averaging 12.7 weeks in duration (ranging from 2 to 52 weeks) yield small but significant increases in PA [32] and that daily text messaging has been associated with increases in PA [99]. However, in the systematic review and meta-analysis citing the duration and effectiveness of web- and text-based interventions, the overwhelming majority of studies were with White non-Latino adults [32]. The Hombres Salduables intervention will provide preliminary effect sizes of a 6 month web- and text-based intervention for diverse Latino men that uses less frequent text messaging than the daily text messaging identified in the PA literature [32,99]. Our findings, if supported via additional research, could help inform the study design for future interventions that involve text messages, including (and perhaps beyond) PA interventions targeted to diverse Latino men.

In addition, achieving the study aims will provide evidence for potential mediators and moderators of PA, which remain underexamined in Latino men. The mediators in this study are based on theoretical constructs (eg, self-efficacy and social support) associated with increases in PA among Latina women and other racial or ethnic subgroups [11-15,28,77]. Although, well-established evidence points to the effects of these mediators on PA, this study provides preliminary evidence as to whether these mediators explain changes in PA among diverse Latino men.

Although the intervention focuses mostly on changing the psychological, behavioral, and economic barriers to PA, we will explore potential moderating effects of individual and environmental factors on PA. At the individual level, we will examine whether important demographics (eg, age, country of origin, marital status, and education), acculturation, or baseline stage of change (based on TTM) impact changes in PA among Latino men. At the neighborhood level, we will explore walkability and social conditions, which have not been reported in PA intervention studies with Latino men. Although many studies explore associations of neighborhood walkability with PA (including studies with Latinos) or as a potential moderator of intervention efficacy [100-104], associations between other neighborhood social conditions and PA remain unanswered. This study will provide preliminary evidence for some of these unanswered questions. For example, this intervention will examine whether perceived neighborhood-based police profiling impacts PA among Latino men. In previous qualitative studies with disparity groups, neighborhood race and gender-based police profiling is cited as a potential barrier to PA for racial or ethnic minority men [89,105]. Consequently, this study seeks to build upon the limited available evidence for Latino men by exploring the potential moderating effects of these individual and environmental variables in a PA-focused pilot RCT.

### Study Limitations and Strengths

Although the strengths of this study are numerous, a few potential challenges exist. Recruitment and retention of Latinos in PA interventions are often challenging [106], and it is possible that there are unforeseen confounding factors that may impact the study results. However, the purpose of this pilot RCT is to determine the feasibility, acceptability, and preliminary efficacy of the intervention. Although contamination is often a concern when implementing interventions, we do not expect this to be a significant problem. If members of the same family participated, they were yoked together. In addition, the PA Facebook group membership was accessible only to participants in the PA intervention, and the web- and text message–based components were tailored to each participant. Despite these potential limitations, the Hombres Saludables study aims to increase PA among diverse Latino men by engaging them in a culturally appropriate, low-cost, and easily accessible internet and cell phone–based intervention. The intervention also provided economic incentives, including a gym membership, to overcome the cost barriers associated with PA. To our knowledge, no studies have included all the combined intervention components that are incorporated into the Hombres Saludables study. We expect that this study will demonstrate preliminary efficacy and lead us to implement a larger RCT in the future. This intervention has the potential to be scalable and to reach and engage a large number of diverse Latino men, a disparity group with respect to PA and related chronic diseases and mental health [1,2,8-10,18].

## Appendix

Multimedia Appendix 1 Screenshots of the Hombres Saludables website.

Multimedia Appendix 2 Peer review of study grant proposal.

## Abbreviations

ANC: Active Neighborhood Checklist
MVPA: moderate-to-vigorous physical activity
PA: physical activity
PAR: physical activity recall
RCT: randomized controlled trial
SCT: social cognitive theory
S-TOFHLA: Short Test of Functional Health Literacy in Adults
TTM: transtheoretical model

## References

[1] (2018). ACSM's guidelines for exercise testing and prescription. 10th ed. American College of Sports Medicine.

[2] (2018). Age-adjusted percent distributions (with standard errors) of participation in leisure-time aerobic and muscle-strengthening activities that meet the 2008 federal physical activity guidelines among adults aged 18 and over, by selected characteristics: United States. U.S.Dept. Of Health and Human Services.

[3] Marquez DX, Neighbors CJ, Bustamante EE (2010). Leisure time and occupational physical activity among racial or ethnic minorities. Med Sci Sports Exerc.

[4] Gay JL, Buchner DM (2014). Ethnic disparities in objectively measured physical activity may be due to occupational activity. Prev Med.

[5] Troiano RP, Berrigan D, Dodd KW, Mâsse LC, Tilert T, McDowell M (2008). Physical activity in the United States measured by accelerometer. Med Sci Sports Exerc.

[6] Tucker JM, Welk GJ, Beyler NK (2011). Physical activity in U.S.: adults compliance with the physical activity guidelines for Americans. Am J Prev Med.

[7] Arredondo EM, Sotres-Alvarez D, Stoutenberg M, Davis SM, Crespo NC, Carnethon MR, Castañeda SF, Isasi CR, Espinoza RA, Daviglus ML, Perez LG, Evenson KR (2016). Physical activity levels in U.S. Latino/Hispanic adults: results from the hispanic community health study/study of Latinos. Am J Prev Med.

[8] National Center for Health Statistics (2018). Normal weight, overweight, and obesity among adults aged 20 and over, by selected characteristics: United States, selected years 1988-1994 through 2013-2016. Health, United States.

[9] (2020). National diabetes statistics report. Centers for Disease Control and Prevention.

[10] Neighbors CJ, Marquez DX, Marcus BH (2008). Leisure-time physical activity disparities among Hispanic subgroups in the United States. Am J Public Health.

[11] Pekmezi D, Dunsiger S, Gans K, Bock B, Gaskins R, Marquez B, Lee C, Neighbors C, Jennings E, Tilkemeier P, Marcus B (2012). Rationale, design, and baseline findings from Seamos Saludables: a randomized controlled trial testing the efficacy of a culturally and linguistically adapted, computer- tailored physical activity intervention for Latinas. Contemp Clin Trials.

[12] Marcus BH, Dunsiger SI, Pekmezi DW, Larsen BA, Bock BC, Gans KM, Marquez B, Morrow KM, Tilkemeier P (2013). The Seamos Saludables study: a randomized controlled physical activity trial of Latinas. Am J Prev Med.

[13] Marcus BH, Dunsiger SI, Pekmezi D, Larsen BA, Marquez B, Bock BC, Gans KM, Morrow KM, Tilkemeier P (2015). Twelve-month physical activity outcomes in Latinas in the Seamos Saludables trial. Am J Prev Med.

[14] Marcus BH, Hartman SJ, Pekmezi D, Dunsiger SI, Linke S, Marquez B, Gans KM, Bock BC, Larsen BA, Rojas C (2015). Using interactive internet technology to promote physical activity in Latinas: rationale, design, and baseline findings of Pasos Hacia La Salud. Contemp Clin Trials.

[15] Marcus BH, Hartman SJ, Larsen BA, Pekmezi D, Dunsiger SI, Linke S, Marquez B, Gans KM, Bock BC, Mendoza-Vasconez AS, Noble ML, Rojas C (2016). Pasos Hacia La Salud: a randomized controlled trial of an internet-delivered physical activity intervention for Latinas. Int J Behav Nutr Phys Act.

[16] Ickes MJ, Sharma M (2012). A systematic review of physical activity interventions in Hispanic adults. J Environ Public Health.

[17] Griffith DM, Bergner EM, Cornish EK, McQueen CM (2018). Physical activity interventions with African Mmerican or Latino men: a systematic review. Am J Mens Health.

[18] El Masri A, Kolt GS, George ES (2019). Physical activity interventions among culturally and linguistically diverse populations: a systematic review. Ethn Health.

[19] Larsen BA, Dunsiger S, Hartman S, Nodora J, Pekmezi DW, Marquez B, Noble M, Rojas C, Marcus BH (2014). Activo: assessing the feasibility of designing and implementing a physical activity intervention for Latino men. Int J of Men's Health.

[20] Beleigoli AM, Andrade AQ, Cançado AG, Paulo MN, Diniz MDFH, Ribeiro AL (2019). Web-based digital health interventions for weight loss and lifestyle habit changes in overweight and obese adults: systematic review and meta-analysis. J Med Internet Res.

[21] Garcia DO, Valdez LA, Aceves B, Bell ML, Humphrey K, Hingle M, McEwen M, Hooker SP (2019). A gender- and culturally sensitive weight loss intervention for Hispanic men: results from the pilot randomized controlled trial. Health Educ Behav.

[22] Larsen BA, Benitez TJ, Mendoza-Vasconez AS, Hartman SJ, Linke SE, Pekmezi DJ, Dunsiger SI, Nodora JN, Gans KM, Marcus BH (2020). Randomized trial of a physical activity intervention for Latino men: Activo. Am J Prev Med.

[23] Redding CA, Prochaska JO, Pallonen UE, Rossi JS, Velicer WF, Rossi SR, Greene GW, Meier KS, Evers KE, Plummer BA, Maddock JE (1999). Transtheoretical individualized multimedia expert systems targeting adolescents' health behaviors. Cogni and Beh Pract.

[24] Prochaska JO, Evers KE, Castle PH, Johnson JL, Prochaska JM, Rula EY, Coberley C, Pope JE (2012). Enhancing multiple domains of well-being by decreasing multiple health risk behaviors: a randomized clinical trial. Popul Health Manag.

[25] Romain AJ, Bortolon C, Gourlan M, Carayol M, Decker E, Lareyre O, Ninot G, Boiché J, Bernard P (2018). Matched or nonmatched interventions based on the transtheoretical model to promote physical activity. A meta-analysis of randomized controlled trials. J Sport Health Sci.

[26] Casper JM, Harrolle MG (2013). Perceptions of constraints to leisure time physical activity among Latinos in Wake County, North Carolina. Am J Health Promot.

[27] Gordon-Larsen P, Nelson MC, Page P, Popkin BM (2006). Inequality in the built environment underlies key health disparities in physical activity and obesity. Pediatrics.

[28] Larsen BA, Noble ML, Murray KE, Marcus BH (2014). Physical activity in Latino men and women. Amer J of Lifestyle Med.

[29] (2019). Internet/broadband fact sheet. Pew Research Center.

[30] (2019). Mobile fact sheet. Pew Research Center.

[31] Perrin A, Turner E (2019). Smartphones help Blacks, Hispanics bridge some but not all digital gaps with Whites. Pew Research Center.

[32] Davies CA, Spence JC, Vandelanotte C, Caperchione CM, Mummery WK (2012). Meta-analysis of internet-delivered interventions to increase physical activity levels. Int J Behav Nutr Phys Act.

[33] Joseph RP, Durant NH, Benitez TJ, Pekmezi DW (2014). Internet-based physical activity interventions. Am J Lifestyle Med.

[34] Jahangiry L, Farhangi MA, Shab-Bidar S, Rezaei F, Pashaei T (2017). Web-based physical activity interventions: a systematic review and meta-analysis of randomized controlled trials. Public Health.

[35] Fanning J, Mullen SP, McAuley E (2012). Increasing physical activity with mobile devices: a meta-analysis. J Med Internet Res.

[36] Hall AK, Cole-Lewis H, Bernhardt JM (2015). Mobile text messaging for health: a systematic review of reviews. Annu Rev Public Health.

[37] Hartman SJ, Dunsiger SI, Bock BC, Larsen BA, Linke S, Pekmezi D, Marquez B, Gans KM, Mendoza-Vasconez AS, Marcus BH (2017). Physical activity maintenance among Spanish-speaking Latinas in a randomized controlled trial of an internet-based intervention. J Behav Med.

[38] Aguirre AC, Ebrahim N, Shea JA (2005). Performance of the English and Spanish S-TOFHLA among publicly insured Medicaid and Medicare patients. Patient Educ Couns.

[39] Parker RM, Baker DW, Williams MV, Nurss JR (1995). The test of functional health literacy in adults: a new instrument for measuring patients' literacy skills. J Gen Intern Med.

[40] Baker DW, Williams MV, Parker RM, Gazmararian JA, Nurss J (1999). Development of a brief test to measure functional health literacy. Patient Educ Couns.

[41] Bandura A (1986). Social foundations of thought and action: a social cognitive theory.

[42] Prochaska JO, DiClemente CC (1983). Stages and processes of self-change of smoking: toward an integrative model of change. J Consult Clin Psychol.

[43] Prochaska J, Redding C, Evers K (2008). The transtheoretical model and stages of change. Health behavior and health education: Theory, research and practice.

[44] Kreuter MW, Lukwago SN, Bucholtz RDDC, Clark EM, Sanders-Thompson V (2003). Achieving cultural appropriateness in health promotion programs: targeted and tailored approaches. Health Educ Behav.

[45] Resnicow K, Baranowski T, Ahluwalia JS, Braithwaite RL (1999). Cultural sensitivity in public health: defined and demystified. Ethn Dis.

[46] Kreuter MW, McClure SM (2004). The role of culture in health communication. Annu Rev Public Health.

[47] Elder JP, Ayala GX, Parra-Medina D, Talavera GA (2009). Health communication in the Latino community: issues and approaches. Annu Rev Public Health.

[48] Sidani S, Guruge S, Miranda J, Ford-Gilboe M, Varcoe C (2010). Cultural adaptation and translation of measures: an integrated method. Res Nurs Health.

[49] Behling O, Law KS (2000). Translating questionnaires and other research instruments: Problems and solutions. Sage Research Methods.

[50] Cella D, Hernandez L, Bonomi AE, Corona M, Vaquero M, Shiomoto G, Baez L (1998). Spanish language translation and initial validation of the functional assessment of cancer therapy quality-of-life instrument. Med Care.

[51] Beaton DE, Bombardier C, Guillemin F, Ferraz MB (2000). Guidelines for the process of cross-cultural adaptation of self-report measures. Spine (Phila Pa 1976).

[52] Harkness J, van DVF, Mohler P (2002). Cross-cultural survey methods. Survey Research Methods & Sampling.

[53] Hunt SM, Bhopal R (2004). Self report in clinical and epidemiological studies with non-English speakers: the challenge of language and culture. J Epidemiol Community Health.

[54] Hofstede G (2003). Culture's consequences: comparing values, behaviors, institutions, and organizations across nations. UNF_RESEARCH.

[55] Valdez LA, Morrill KE, Griffith DM, Lindberg NM, Hooker SP, Garcia DO (2019). Mexican origin hispanic men's perspectives of physical activity-related health behaviors. Am J Mens Health.

[56] Gavarkovs AG, Burke SM, Petrella RJ (2017). The physical activity-related barriers and facilitators perceived by men living in rural communities. Am J Mens Health.

[57] Payán DD, Sloane DC, Illum J, Lewis LB (2019). Intrapersonal and environmental barriers to physical activity among Blacks and Latinos. J Nutr Educ Behav.

[58] Stults-Kolehmainen MA, Sinha R (2014). The effects of stress on physical activity and exercise. Sports Med.

[59] Pechmann C, Phillips C, Calder D, Prochaska JJ (2020). Facebook recruitment using zip codes to improve diversity in health research: longitudinal observational study. J Med Internet Res.

[60] McCarthy E, Mazza D (2019). Cost and effectiveness of using facebook advertising to recruit young women for research: prefer (contraceptive preferences study) experience. J Med Internet Res.

[61] Akers L, Gordon JS (2018). Using Facebook for large-scale online randomized clinical trial recruitment: effective advertising strategies. J Med Internet Res.

[62] Sallis JF, Haskell WL, Wood PD, Fortmann SP, Rogers T, Blair SN, Paffenbarger RS (1985). Physical activity assessment methodology in the Five-City Project. Am J Epidemiol.

[63] Blair SN, Haskell WL, Ho P, Paffenbarger RS, Vranizan KM, Farquhar JW, Wood PD (1985). Assessment of habitual physical activity by a seven-day recall in a community survey and controlled experiments. Am J Epidemiol.

[64] Pekmezi DW, Neighbors CJ, Lee CS, Gans KM, Bock BC, Morrow KM, Marquez B, Dunsiger S, Marcus BH (2009). A culturally adapted physical activity intervention for Latinas: a randomized controlled trial. Am J Prev Med.

[65] Norris AE, Ford K, Bova CA (2016). Psychometrics of a brief acculturation scale for Hispanics in a probability sample of urban Hispanic adolescents and young adults. Hispanic J of Beh Sci.

[66] Freedson PS, Melanson E, Sirard J (1998). Calibration of the Computer Science and Applications, Inc. Accelerometer. Med Sci Sports Exerc.

[67] White DK, Gabriel KP, Kim Y, Lewis CE, Sternfeld B (2015). Do short spurts of physical activity benefit cardiovascular health? The CARDIA study. Med Sci Sports Exerc.

[68] Piercy KL, Troiano RP, Ballard RM, Carlson SA, Fulton JE, Galuska DA, George SM, Olson RD (2018). The physical activity guidelines for Americans. JAMA.

[69] Janz KF (1994). Validation of the CSA accelerometer for assessing children's physical activity. Med Sci Sports Exerc.

[70] Melanson EL, Freedson PS (1995). Validity of the Computer Science and Applications, Inc. (CSA) activity monitor. Med Sci Sports Exerc.

[71] Prince SA, Adamo KB, Hamel ME, Hardt J, Connor Gorber S, Tremblay M (2008). A comparison of direct versus self-report measures for assessing physical activity in adults: a systematic review. Int J Behav Nutr Phys Act.

[72] Hayden-Wade HA, Coleman KJ, Sallis JF, Armstrong C (2003). Validation of the telephone and in-person interview versions of the 7-day PAR. Med Sci Sports Exerc.

[73] Leenders NY, Sherman WM, Nagaraja HN, Kien CL (2001). Evaluation of methods to assess physical activity in free-living conditions. Med Sci Sports Exerc.

[74] Dunn AL, Garcia ME, Marcus BH, Kampert JB, Kohl HW, Blair SN (1998). Six-month physical activity and fitness changes in Project Active, a randomized trial. Med Sci Sports Exerc.

[75] Dunn AL, Marcus BH, Kampert JB, Garcia ME, Kohl HW, Blair SN (1999). Comparison of lifestyle and structured interventions to increase physical activity and cardiorespiratory fitness: a randomized trial. JAMA.

[76] Rauh MJ, Hovell MF, Hofstetter CR, Sallis JF, Gleghorn A (1992). Reliability and validity of self-reported physical activity in Latinos. Int J Epidemiol.

[77] Marcus BH, Selby VC, Niaura RS, Rossi JS (1992). Self-efficacy and the stages of exercise behavior change. Res Q Exerc Sport.

[78] Bock BC, Marcus BH, Pinto BM, Forsyth LH (2001). Maintenance of physical activity following an individualized motivationally tailored intervention. Ann Behav Med.

[79] Marcus BH, Rossi JS, Selby VC, Niaura RS, Abrams DB (1992). The stages and processes of exercise adoption and maintenance in a worksite sample. Health Psychol.

[80] Sallis JF, Grossman RM, Pinski RB, Patterson TL, Nader PR (1987). The development of scales to measure social support for diet and exercise behaviors. Prev Med.

[81] Kendzierski D, DeCarlo KJ (1991). Physical activity enjoyment scale: two validation studies. J of Sport and Exercise Psychol.

[82] Cohen S, Kamarck T, Mermelstein R (1983). A global measure of perceived stress. J Health Soc Behav.

[83] Cohen S, Inpacapan S, Oskamp S (1988). Perceived stress in a probability sample of the United States. The social psychology of health.

[84] Skevington SM, Lotfy M, O'Connell KA, WHOQOL Group (2004). The World Health Organization's WHOQOL-BREF quality of life assessment: psychometric properties and results of the international field trial. A report from the WHOQOL group. Qual Life Res.

[85] (2015). California health interview survey (CHIS) 2013-2014 adult questionnaire. UCLA Center for Health Policy Research.

[86] Mujahid MS, Diez Roux AV, Morenoff JD, Raghunathan T (2007). Assessing the measurement properties of neighborhood scales: from psychometrics to ecometrics. Am J Epidemiol.

[87] Schuck AM, Rosenbaum DP, Hawkins DF (2008). The influence of race/ethnicity, social class, and neighborhood context on residents' attitudes toward the police. Police Quarterly.

[88] Schuck AM, Rosenbaum DP (2005). Global and neighborhood attitudes toward the police: differentiation by race, ethnicity and type of contact. J Quant Criminol.

[89] Dulin-Keita A, Hannon L, Buys D, Casazza K, Clay O (2016). Surrounding community residents' expectations of HOPE VI for their community, health and physical activity. J Community Pract.

[90] Odgers CL, Caspi A, Bates CJ, Sampson RJ, Moffitt TE (2012). Systematic social observation of children's neighborhoods using Google Street View: a reliable and cost-effective method. J Child Psychol Psychiatry.

[91] Kelly CM, Wilson JS, Baker EA, Miller DK, Schootman M (2013). Using Google Street View to audit the built environment: inter-rater reliability results. Ann Behav Med.

[92] Wilson J, Kelly C (2011). Navigating Google street view: a guide to conducting audits of the built environment using Google street view. Active Living Research.

[93] Hoehner CM, Ivy A, Ramirez LKB, Handy S, Brownson RC (2007). Active neighborhood checklist: a user-friendly and reliable tool for assessing activity friendliness. Am J Health Promot.

[94] Wilson JS, Kelly CM, Schootman M, Baker EA, Banerjee A, Clennin M, Miller DK (2012). Assessing the built environment using omnidirectional imagery. Am J Prev Med.

[95] Subar AF, Thompson FE, Kipnis V, Midthune D, Hurwitz P, McNutt S, McIntosh A, Rosenfeld S (2001). Comparative validation of the Block, Willett, and National Cancer Institute food frequency questionnaires : the Eating at America's Table Study. Am J Epidemiol.

[96] Booth FW, Roberts CK, Thyfault JP, Ruegsegger GN, Toedebusch RG (2017). Role of inactivity in chronic diseases: evolutionary insight and pathophysiological mechanisms. Physiol Rev.

[97] Booth FW, Roberts CK, Laye MJ (2012). Lack of exercise is a major cause of chronic diseases. Compr Physiol.

[98] Gonzalez M, Sanders-Jackson A, Wright T (2019). Web-based health information technology: access among latinos varies by subgroup affiliation. J Med Internet Res.

[99] Shaw R, Bosworth H (2012). Short message service (SMS) text messaging as an intervention medium for weight loss: a literature review. Health Informatics J.

[100] Perez LG, Kerr J, Sallis JF, Slymen D, McKenzie TL, Elder JP, Arredondo EM (2018). Perceived neighborhood environmental factors that maximize the effectiveness of a multilevel intervention promoting physical activity among latinas. Am J Health Promot.

[101] Murillo R, Reesor LM, Hernandez DC, Obasi EM (2019). Neighborhood Walkability and Aerobic Physical Activity among Latinos. Am J Health Behav.

[102] Barnes R, Giles-Corti B, Bauman A, Rosenberg M, Bull FC, Leavy JE (2013). Does neighbourhood walkability moderate the effects of mass media communication strategies to promote regular physical activity?. Ann Behav Med.

[103] Kerr J, Norman GJ, Adams MA, Ryan S, Frank L, Sallis JF, Calfas KJ, Patrick K (2010). Do neighborhood environments moderate the effect of physical activity lifestyle interventions in adults?. Health and Place.

[104] Hajna S, Ross NA, Brazeau A, Bélisle P, Joseph L, Dasgupta K (2015). Associations between neighbourhood walkability and daily steps in adults: a systematic review and meta-analysis. BMC Public Health.

[105] Ray R (2017). Black people don't exercise in my neighborhood: perceived racial composition and leisure-time physical activity among middle class blacks and whites. Soc Sci Res.

[106] Eakin EG, Bull SS, Riley K, Reeves MM, Gutierrez S, McLaughlin P (2007). Recruitment and retention of Latinos in a primary care-based physical activity and diet trial: the resources for health study. Health Educ Res.

