# Supplementary figures and images for: A Tailored Web- and Text-Based Intervention to Increase Physical Activity for Latino Men: Protocol for a Randomized Controlled Feasibility Trial
Source: JMIR Res Protoc. 2021 Jan 29;10(1):e23690. doi: 10.2196/23690 (PMC7880809; doi:10.2196/23690)

Multimedia Appendix 2. Screenshots of the Website


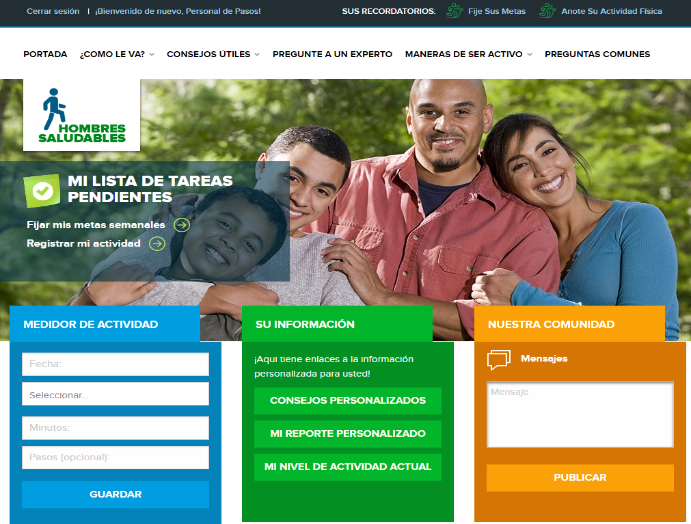


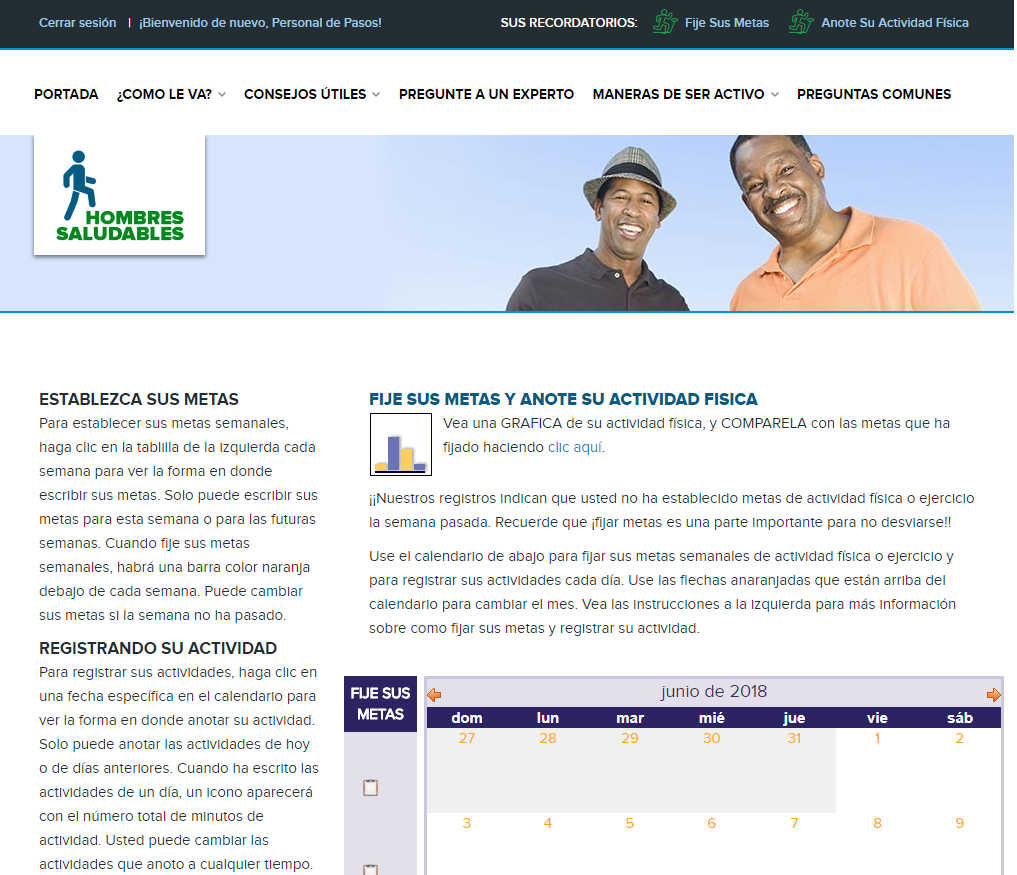


##
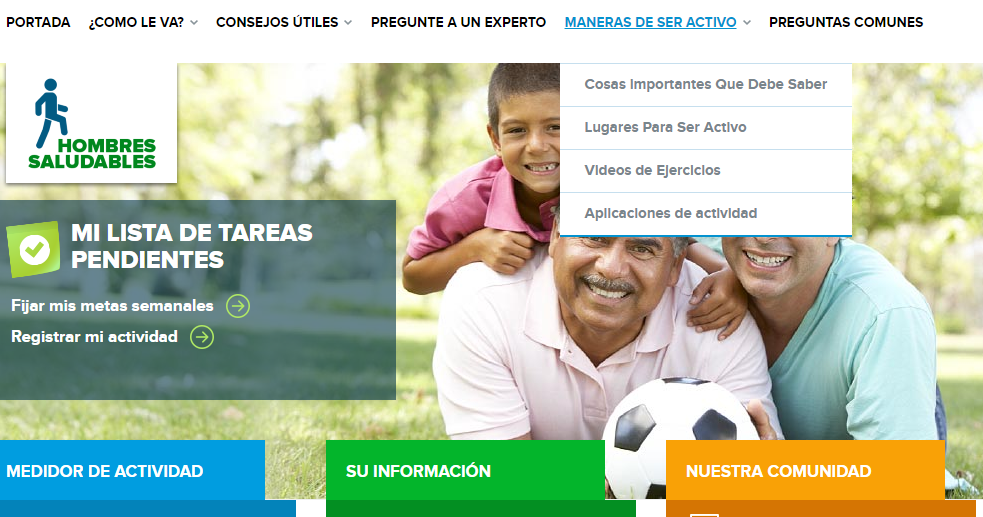

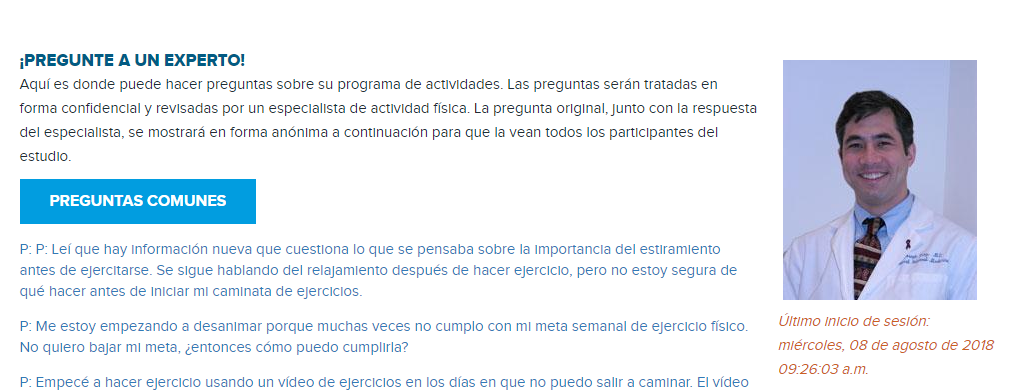


##
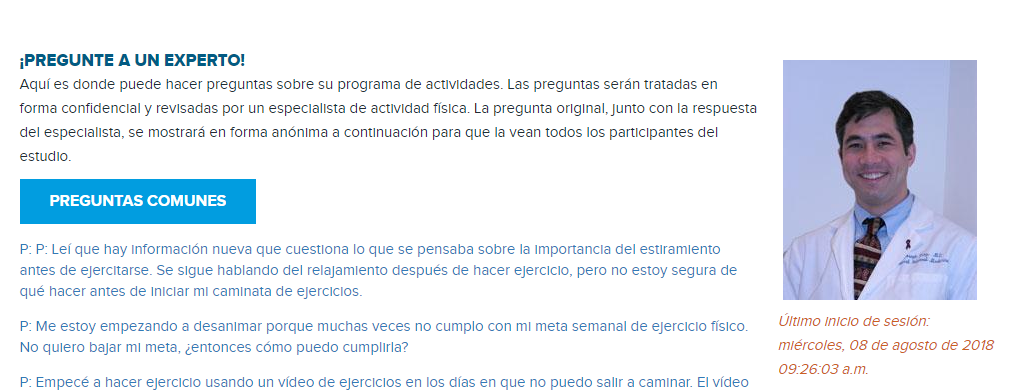

Supplement: Multimedia Appendix 1 [file resprot_v10i1e23690_app1.docx]
