# Supplementary material for: A Tailored Web- and Text-Based Intervention to Increase Physical Activity for Latino Men: Protocol for a Randomized Controlled Feasibility Trial
Source: JMIR Res Protoc. 2021 Jan 29;10(1):e23690. doi: 10.2196/23690 (PMC7880809; doi:10.2196/23690)
Supplement: Multimedia Appendix 2 [file resprot_v10i1e23690_app2.pdf]

SUMMARY STATEMENT
(Privileged Communication)

PROGRAM CONTACT:
Charlotte Pratt
(301) 435-0382
prattc@nhlbi.nih.gov

Release Date: 04/20/2016 03:36 PM
Revised Date:

Application Number: 1 R34 HL128067-01A1

Principal Investigators (Listed Alphabetically):

GANS, KIM M. (Contact)
KEITA, AKILAH JOYCE
MARCUS, BESS HYA

Applicant Organization: UNIVERSITY OF CONNECTICUT STORRS

Review Group: CLTR (MA)
Clinical Trials Review Committee

Meeting Date: 03/03/2016
Council: MAY 2016
Requested Start: 07/01/2016
RFA/PA: PAR13-002
PCC: HHCG N

Project Title: Physical Activity in Latino Men Through Tailoring: Hombres Saludables

SRG Action: Impact Score:13
Next Steps: Visit http://grants.nih.gov/grants/next_steps.htm
Human Subjects: 30-Human subjects involved - Certified, no SRG concerns
Animal Subjects: 10-No live vertebrate animals involved for competing appl.
Gender: 3A-Only men, scientifically acceptable
Minority: 2A-Only minorities, scientifically acceptable
Children: 1A-Both Children and Adults, scientifically acceptable
Clinical Research - not NIH-defined Phase III Trial

Project Year	Direct Costs Requested	Estimated Total Cost
1	175,000	290,805
2	150,000	249,261
3	125,000	207,718
TOTAL	450,000	747,783

ADMINISTRATIVE BUDGET NOTE: The budget shown is the requested budget and has not been adjusted to reflect any recommendations made by reviewers. If an award is planned, the costs will be calculated by Institute grants management staff based on the recommendations outlined below in the COMMITTEE BUDGET RECOMMENDATIONS section.

1R34HL128067-01A1 GANS, KIM (contact)
KEITA, AKILAH
MARCUS, BESS

RESUME AND SUMMARY OF DISCUSSION: This is a revised R34 pilot trial of a physical activity program in Latino men. The application has several strengths. The revised application is very responsive to previous critiques. The significance of the application is high, as an evidence-based physical activity program in Latino men is needed. The application was considered innovative in terms of the target study population and the plans to evaluate the potential for heterogeneity of treatment effects. The approach proposed in the application builds on similar work in Latino women, employs a mixed methods approach with additional qualitative work to inform the study design of the pilot clinical trial tailored to the study population. The investigator team is strong, with an appropriate multi-PI plan, and the research environment is appropriate for the proposed studies. The only minor weakness was that it was unclear if Mexican men will be excluded, and if so, that this exclusion could limit the generalizability of study results. Overall, the plans regarding the recruitment of minorities, women, and children were considered scientifically acceptable.

DESCRIPTION (provided by applicant): Physical inactivity is a physical inactivity than risk factor for many important chronic diseases. Latino men report higher rates of non-Latinos are less likely to meet PA guidelines and are disproportionately affected by PA-related health conditions, such as obesity and diabetes. Most Latino PA interventions have focused on women; the vast majority excluded men and none specifically targeted men. Moreover interventions that included men focused mostly on Mexican American only. Thus, there is an urgent need for effective PA interventions with diverse populations of Latino men. NIH has specifically called for research studies to focus on the development and testing of culturally appropriate health-promoting interventions to reduce health disparities among racially and ethnically diverse men. Our research team has 25 years of experience developing, implementing and evaluating individually tailored, theory-based, computer-driven PA interventions including `Seamos Saludables' (in RI) and `Pasos' (in CA), culturally and linguistically adapted, individually- tailored print (Seamos) and web-based (Pasos) interventions for a diverse population of sedentary Latina women that were successful in increasing PA in Latina women. We then conducted formative interviews with Latino men, which confirmed that an intervention would be well-received by them. However, substantial content modifications were needed, so we adapted the print intervention and tested it in a demonstration trial with 10 Mexican American men. After 12 weeks, participants reported a significant increase (p=.03) in mean minutes of PA from 1.50 min/week at baseline to 125.70 min/week at 12 weeks with 100% retention and expressed enthusiasm for the program. However, less than half of the men achieved the PA guidelines of 150 minutes/week. Thus, further enhancements appear to be needed. Follow-up interviews indicated that the intervention was well received, but the men felt that they needed greater accountability, more contact with staff and other participants, briefer and more frequent information, more updates on PA opportunities, and ideas for addressing environmental barriers. The men specifically mentioned interest in web and/or text-based interventions to meet these needs. Thus, we will conduct formative research with a diverse population of Latino men in RI to inform the adaptation of the Pasos web-based intervention that was developed for Latina women, enhance it with new cell phone/text-based intervention components, and ensure that it is culturally appropriate for Latino men of Caribbean, South and Central American origin. We will then conduct a pilot study to evaluate its feasibility, acceptability, and preliminary efficacy with a diverse population of Latino men in RI. In the pilot study, we will also explore potential moderators of treatment effects including demographics, acculturation, and environmental variables such as the neighborhood built, social and economic environments. The results of this pilot study will inform a future randomized controlled trial with Latino men to increase PA.

PUBLIC HEALTH RELEVANCE: Latino men report particularly low rates of physical activity (PA) and are disproportionately burdened by health conditions related to sedentary lifestyle. The proposed research will build on a series of previous successful studies to develop an individually tailored intervention using a combination of web and texting that is culturally and linguistically appropriate for a diverse population of Latino men and addresses their PA barriers and preferences. We will conduct focus groups with a diverse population of Latino men to ensure that the intervention is culturally appropriate for Latino men of Caribbean, South and Central American origin and to adapt/develop the new intervention components. Then we will evaluate the intervention's feasibility, acceptability, and preliminary efficacy in a pilot study with 50 diverse Latino men.

CRITIQUE 1:

Significance: 1
Investigator(s): 1
Innovation: 2
Approach: 1
Environment: 1

Overall Impact:
The aim of the revised study is to develop a physical activity (PA) intervention tailored to the needs of Latino men and which could be potentially scalable if effective. The PI has been very responsive to the critiques and the program of research is substantially improved. In particular, with some changes to the methodology, the application now is able to ask a theoretically and methodologically important question: How does the physical environment, including access to recreational activities, affect the outcome of individual-level interventions focused primarily on increasing motivation and shaping self-regulatory skills. The person X environment interactions which can now be investigated will yield information which could be of value for a wide range of lifestyle interventions. The research team is excellent; they have a history of successful work with both PA interventions and with this population. The intervention itself is based on a program that has been successfully used with Latina women which they have modified in some initial testing to meet the needs of men. The intervention emerges from a solid theoretical foundation; with intervention components based on Social Cognitive theory and trans-theoretical models. Even the content of the text messaging is based on empirically validated behavior change principles related to normative and aspirational messaging, problems solving, etc. The control condition (NIH materials on health) is now appropriate. Portions of the intervention will now be delivered via the web. Good information is provided on the potential R01 which would follow this study.

1. Significance:
Strengths
- Low levels of PA are a significant risk factor for a wide variety of chronic illness. Low PA is a specific problem for Latino men.
- There have been very few targeted interventions for Latino men, and developing these interventions is a recognized need.
- Self-managed PA intervention is relatively low cost and scalable and could create population level differences in health outcomes.
- Estimating the ways in which neighborhood factors influence outcomes could provide additional needed insight into environmental barriers to PA. This information could help identify resources needed to facilitate self-management. This intervention, like most current lifestyle interventions is focused on improving individual-level motivation and self-regulatory skills. However, very little is known about the degree to which barriers in the social and physical environment influence the effectiveness of individual-level motivational/skills building interventions. This study now has a much more detailed evaluation of the barriers and of the physical environment. This will allow the investigators to understand the degree to which these barriers (e.g. distance from parkland

or other recreational facilities, etc.) are surmountable by individual-level interventions to improve motivation and skills.

Weaknesses

- It would have been stronger if the PI were able to use a stratified sampling method, such that half the intervention and control participants could come from neighborhoods with high versus low physical resources. However, this may not be feasible and could be a goal for a future study.
- It is unclear if the PI anticipates including Mexican-American men. If so, how will group (i.e., Mexican vs. Columbian vs. Puerto Rican, etc.) differences in the drivers of PA be accounted for in the conceptualization of the study and analyses of the outcomes.

2. Investigator(s):

Strengths

- This is a very strong research team with good experience in community based research. This is difficult research to do, and the researchers are clearly very experienced in working with this population. Good coordination plans.

Weaknesses

- None noted.

3. Innovation:

Strengths

- The detailed assessment of barriers, both personal and environmental, substantially improves the innovation of this application. The target population is innovative. The content of the text messaging is innovative – a theory-based improvement on the usual reminders to complete the activity. The use of the web to target interventions for Latino men is still moderately innovative.

Weaknesses

- Self-managed PA interventions are not innovative at this point.

4. Approach:

Strengths

- Good recruitment strategies.
- Good measures of PA, health literacy, acculturation. Thorough, well documented.
- Good procedures for screening, pre-test assessments, follow-up.
- Good application of empirically validated strategies to text messaging and intervention content.
- Good retention procedures.
- Reasonable analytic strategy.
- Improved control condition.
- Better measures of personal and environmental barriers.

Weaknesses

- None noted.

5. Environment:

Strengths

- Strong environment with all needed resources.

Weaknesses

- None noted.

Protections for Human Subjects:

Acceptable Risks and/or Adequate Protections

- Thoughtful plan.

Data and Safety Monitoring Plan (Applicable for Clinical Trials Only):
Acceptable

- DSMB added.

Inclusion of Women, Minorities and Children:

- Sex/Gender: Distribution justified scientifically.
- Race/Ethnicity: Distribution justified scientifically.
- Inclusion/Exclusion of Children under 21: Including ages < 21 justified scientifically.
- Will include 18-21 year olds.

CRITIQUE 2:

Significance: 3
Investigator(s): 1
Innovation: 3
Approach: 2
Environment: 1

Overall Impact:
This is a resubmission of a application to study the development and pilot study of an intervention to study web and text based interventions targeting increasing physical activity in Latino Men. This is a similar approach to other studies designed to study Latino women. There are 2 primary aims to 1) develop the intervention using 6 focus groups (8-10 men) which will provide substantive feedback on specific needs for this population; 2) Pilot a small trial in 50 Latino men primarily of Puerto Rican, Dominican and Colombian descent using the web/phone based intervention over 6 months. The participants will be seen at baseline and 6 months with monthly assessments by mail. Participants will receive text messages to increase physical activity. An accelerometer will be used to assess activity at baseline and 6 months which will be worn over 7 days. They will use a pedometer daily and track their activity which will be entered online. Several questionnaires will be administered throughout to assess behavioral change (decisional balance, cognitive/behavioral change process, and self-efficacy). In addition environmental factors will be assessed. Other data will be collected to qualify the overall results such as demographics, acculturation, consumer satisfaction, functional health literacy and 7 day physical activity tracking. The impact of this application is that it will provide the necessary information to conduct a larger more definitive trial in this population of Latino men. This type of targeted intervention has not been done for Latino men in the past in whom the risk of CVD, Diabetes and other disease which are clearly affected by physical inactivity. This is a mirror of studies done in Latino women and similar to a trial ongoing in Mexican men in San Diego.

1. Significance:
Strengths

- The significance of the study is that it targets an at risk group who have not been studied in the past.
- This project will advance the field of lifestyle modification for culturally specific groups.

Weaknesses

- None noted.

2. Investigator(s):
Strengths

- The investigative team is well prepared to conduct this project.

Weaknesses
- None noted.

3. Innovation:
Strengths
- The innovation of this application is the use of the maps software to provide the participants information on public parks, walking/jogging trails/public transportation to the participants in the study.

Weaknesses
- None noted.

4. Approach:
Strengths
- The approach has a detailed approach to evaluating focus group information which will be incorporated into the intervention.
- The addition of the web-based approach is a great addition. It is a direct response to previous review. It has also been tested in PASOS with Latina Women.
- The addition of local mapped areas for physical activity in the local area for each participant is a good way to decrease the barrier to exercise.
- The 2 phase approach is an excellent way to collect data to prepare for a larger more definitive trial.
- The planned study will determine the feasibility and planning of intervention for the main trial. There was a limited weight loss in the pilot study. It is not clearly identified how the trial will enhance this weight loss.
- The texting approach is detailed and specific.
- The Pedometer is a good measure of physical activity.
- They will provide a small but likely impactful reimbursement to help the participants in small way.

Weaknesses
- It is not clear in the application why this intervention is planned in Latino men excluding Mexican men.
- Is there an exclusion criterion for men of Mexican descent? Why only Colombian, Puerto Rican, and Dominican descent? What about other Latino men?

5. Environment:
Strengths
- Excellent environment.

Weaknesses
- None noted.

Protections for Human Subjects:
Acceptable Risks and/or Adequate Protections

Data and Safety Monitoring Plan (Applicable for Clinical Trials Only):
Acceptable

Inclusion of Women, Minorities and Children:
- Sex/Gender: Distribution justified scientifically.
- Race/Ethnicity: Distribution justified scientifically.
- Inclusion/Exclusion of Children under 21: Excluding ages < 21 justified scientifically.

- It is important to clarify whether Hispanic men of Mexican descent are included or not. If the thought is that this group is not a target of the intervention then it may be appropriate to exclude them.

Resource Sharing Plans:
Acceptable

Budget and Period of Support:
Recommend as Requested.

CRITIQUE 3:

Significance: 1
Investigator(s): 1
Innovation: 1
Approach: 1
Environment: 1

1. Significance:
Strengths
- Latinos are less likely to meet PA guidelines and disproportionately affected by PA-related health conditions than other racial/ethnic groups.
- Most PA interventions have targeted women and none have targeted Latino men.
- Examining the way environmental barriers may contribute to inactivity is important.
- Addresses a call from NIH to focus on men.

Weaknesses
- None noted.

2. Investigator(s):
Strengths
- The investigative team is strong and has experience working together.
- The investigative team has significant experience working with the proposed population and in the proposed area.
- The addition of Dr. Dulin-Keita's expertise in neighborhood risk environments is a strength.
- MPI plan is appropriate.

Weaknesses
- None noted.

3. Innovation:
Strengths
- The targeting of Latino men for this intervention is innovative.
- Use of a diverse population of Latinos.
- Exploring the moderating effects of neighborhood and built social contexts.

Weaknesses
- None noted.

4. Approach:
Strengths
- Methodological concerns from previous reviews addressed.
- Mixed method design.

- Qualitative work to inform the intervention design.
- Well described intervention based on a successful intervention for women.
- Good pilot data.
- Exploration of treatment modifiers.
- Detailed information about future clinical trial and decision rules for whether or not to proceed.
- Builds upon previous experience and successful interventions.
- Good use of a community advisory board.

Weaknesses

- Physical activity and exercise trials often have issues with dropouts in the control condition. There is no discussion of this issue.

5. Environment:

Strengths

- Excellent environment for the study and strong support from the community.
- Needed resources are available.

Weaknesses

- None noted.

Protections for Human Subjects:

Acceptable Risks and/or Adequate Protections

- Well outlined plan and previous concerns about how the texting would work in terms of participant private information have been addressed.

Data and Safety Monitoring Plan (Applicable for Clinical Trials Only):

Acceptable

- Needs more detail -- it is unclear whether the DSMB will meet together or review these items separately as there is no mention of meetings only reviewing reports. DSMB should include a statistician.

Inclusion of Women, Minorities and Children:

- Sex/Gender: Distribution justified scientifically.
- Race/Ethnicity: Distribution justified scientifically.
- Inclusion/Exclusion of Children under 21: Including ages < 21 justified scientifically.
- Including adolescents 18-21.

Budget and Period of Support:

Recommend as Requested.

THE FOLLOWING SECTIONS WERE PREPARED BY THE SCIENTIFIC REVIEW OFFICER TO SUMMARIZE THE OUTCOME OF DISCUSSIONS OF THE REVIEW COMMITTEE, OR REVIEWERS' WRITTEN CRITIQUES, ON THE FOLLOWING ISSUES:

PROTECTION OF HUMAN SUBJECTS (Resume): ACCEPTABLE

Protection of human subjects was considered acceptable. However, the committee recommended more details regarding the meeting schedule for the data safety monitoring board (DSMB) and some consideration about whether to include a statistician in the DSMB.

INCLUSION OF WOMEN PLAN (Resume): ACCEPTABLE

The project does not involve women.

INCLUSION OF MINORITIES PLAN (Resume): ACCEPTABLE
Only minorities – Latino men.

INCLUSION OF CHILDREN PLAN (Resume): ACCEPTABLE
Technically includes children (18-21); reviewers found plans acceptable.

COMMITTEE BUDGET RECOMMENDATIONS: The budget was recommended as requested.

Footnotes for 1 R34 HL128067-01A1; PI Name: Gans, Kim M.

NIH has modified its policy regarding the receipt of resubmissions (amended applications). See Guide Notice NOT-OD-14-074 at http://grants.nih.gov/grants/guide/notice-files/NOT-OD-14-074.html. The impact/priority score is calculated after discussion of an application by averaging the overall scores (1-9) given by all voting reviewers on the committee and multiplying by 10. The criterion scores are submitted prior to the meeting by the individual reviewers assigned to an application, and are not discussed specifically at the review meeting or calculated into the overall impact score. Some applications also receive a percentile ranking. For details on the review process, see http://grants.nih.gov/grants/peer_review_process.htm#scoring.

MEETING ROSTER
Clinical Trials Review Committee
Heart, Lung, and Blood Initial Review Group
NATIONAL HEART, LUNG, AND BLOOD INSTITUTE

CLTR (MA)
03/03/2016 - 03/04/2016

CHAIRPERSON(S)

KRISHNAN, JERRY A., PHD, MD
PROFESSOR OF MEDICINE AND PUBLIC HEALTH
OFFICE OF THE VICE PRESIDENT FOR HEALTH AFFAIRS
UNIVERSITY OF ILLINOIS HOSPITAL
AND HEALTH SCIENCES SYSTEM
CHICAGO, IL 60612

MEMBERS

ALLEN, CARL E, MD, PHD *
ASSISTANT PROFESSOR
DEPARTMENT OF PEDIATRICS
BAYLOR COLLEGE OF MEDICINE
HOUSTON, TX 77030

ATAGA, KENNETH I, MD, MBBS
PROFESSOR
DIVISION OF HEMATOLOGY/ONCOLOGY
DEPARTMENT OF MEDICINE
UNIVERSITY OF NORTH CAROLINA AT CHAPEL HILL
CHAPEL HILL, NC 27514

BENTLEY-LEWIS, RHONDA, MD *
ASSISTANT PROFESSOR
DEPARTMENT OF MEDICINE
HARVARD MEDICAL SCHOOL
BOSTON, MA 02114

BERTONI, ALAIN GERALD, MPH, MD
PROFESSOR AND DEPARTMENT CHAIR
DEPARTMENT OF EPIDEMIOLOGY AND PREVENTION
WAKE FOREST SCHOOL OF MEDICINE
WINSTON-SALEM, NC 27157

BIRGERSDOTTER-GREEN, ULRIKA M, MD *
PROFESSOR OF MEDICINE
DIVISION OF CARDIOVASCULAR MEDICINE
UNIVERSITY OF CALIFORNIA AT SAN DIEGO
SAN DIEGO, CA 92103-8411

BLACKSTONE, EUGENE HUBERT, MD
PROFESSOR
DEPARTMENT THORACIC AND CARDIOVASCULAR
SURGERY
HEART AND VASCULAR INSTITUTE
CLEVELAND CLINIC LERNER COLLEGE OF MEDICINE
CLEVELAND, OH 44195

BOLLARD, CATHERINE MARY, MBBS, MD *
PROFESSOR
DEPARTMENT OF ALLERGY AND IMMUNOLOGY
CHILDREN'S NATIONAL MEDICAL CENTER
WASHINGTON, DC 20010

BRONDOLO, ELIZABETH N, PHD
PROFESSOR
DEPARTMENT OF PSYCHOLOGY
ST. JOHN'S UNIVERSITY
QUEENS, NY 11439

CAIRNS, CHARLES B., MD *
PROFESSOR OF EMERGENCY MEDICINE
DEPARTMENT OF MEDICINE ADMINISTRATION
UNIVERSITY OF ARIZONA HEALTH SCIENCES CENTER
TUCSON, AZ 85724

CASTRO, MARIO, MD
PROFESSOR
DIVISION OF PULMONARY AND CRITICAL CARE MEDICINE
DEPARTMENT OF MEDICINE
SCHOOL OF MEDICINE
WASHINGTON UNIVERSITY
ST. LOUIS, MO 63110

CHOUDHRY, NITEESH K, PHD, MD
ASSOCIATE PROFESSOR
DIVISION OF PHARMACOEPIDEMIOLOGY
AND PHARMACOECONOMICS
DEPARTMENT OF MEDICINE
BRIGHAM AND WOMEN'S HOSPITAL
BOSTON, MA 02120

CRARY, SHELLEY E., MD *
ASSOCIATE PROFESSOR
DEPARTMENT OF PEDIATRICS
UNIVERSITY OF ARKANSAS
LITTLE ROCK, AK 72202

CURLEY, MARTHA AQ, RN, PHD
ELLEN AND ROBERT KAPITO PROFESSOR IN NURSING
SCIENCE
SCHOOL OF NURSING
UNIVERSITY OF PENNSYLVANIA
PHILADELPHIA, PA 19104

FOULKES, MARY A, MPH, PHD
RESEARCH PROFESSOR
DEPARTMENT OF EPIDEMIOLOGY AND BIOSTATISTICS
GEORGE WASHINGTON UNIVERSITY
ROCKVILLE, MD 20852

GERALD, LYNN B, PHD
PROFESSOR, ASSOCIATE DEAN FOR RESEARCH, AND
CANYON RANCH ENDOWED CHAIR
HEALTH PROMOTION SCIENCES DIVISION
MEL AND ENID ZUCKERMAN
COLLEGE OF PUBLIC HEALTH
UNIVERSITY OF ARIZONA
TUCSON, AZ 85724

HORAN, JOHN THOMAS, MD *
ASSOCIATE PROFESSOR
DEPARTMENT OF PEDIATRICS
EMORY UNIVERSITY SCHOOL OF MEDICINE
ATLANTA, GA 30322

JEAN-LOUIS, GIRARDIN, PHD *
PROFESSOR OF POPULATION HEALTH AND PSYCHIATRY
DEPARTMENT OF POPULATION HEALTH
NEW YORK UNIVERSITY SCHOOL OF MEDICINE
NEW YORK, NY 10016

KANER, ROBERT J, MD *
ASSOCIATE PROFESSOR
NEW YORK PRESBYTERIAN HOSPITAL
WEILL CORNELL MEDICAL COLLEGE
NEW YORK, NY 10021

KELSEY, SHERYL F, PHD
PROFESSOR
DEPARTMENT OF EPIDEMIOLOGY
GRADUATE SCHOOL OF PUBLIC HEALTH
UNIVERSITY OF PITTSBURGH
PITTSBURGH, PA 15261

KIM, KYUNGMANN, PHD
PROFESSOR
DEPARTMENT OF BIOSTATISTICS
AND MEDICAL INFORMATICS AND STATISTICS
UNIVERSITY OF WISCONSIN
MADISON, WI 53792

LUO, SHENG, PHD *
ASSISTANT PROFESSOR
DEPARTMENT OF BIOSTATISTICS
UNIVERSITY OF TEXAS HEALTH SCIENCES CENTER
SCHOOL OF PUBLIC HEALTH
HOUSTON, TX 77030

MARK, DANIEL B, MPH, MD
PROFESSOR OF MEDICINE
DUKE CLINICAL RESEARCH INSTITUTE
DUKE UNIVERSITY MEDICAL CENTER
DURHAM, NC 27715

MINICH, L. LUANN, MD
PROFESSOR
DEPARTMENT OF PEDIATRICS
UNIVERSITY OF UTAH
PRIMARY CHILDREN'S MEDICAL CENTER
SALT LAKE CITY, UT 84113

MOKHLESI, BABAK, MD *
PROFESSOR OF MEDICINE
SECTION OF PULMONARY AND CRITICAL CARE
UNIVERSITY OF CHICAGO MEDICAL CENTER
CHICAGO, IL 60637

NESBITT, SHAWNA D, MD
ASSOCIATE PROFESSOR
DEPARTMENT OF INTERNAL MEDICINE
UNIVERSITY OF TEXAS
SOUTHWESTERN MEDICAL CENTER
DALLAS, TX 75390

PARKER, MARGARET, MD *
DIRECTOR, PEDIATRIC INTENSIVE CARE UNIT
DEPARTMENT OF MEDICINE AND ANESTHESIA
STONY BROOK UNIVERSITY
STONY BROOK, NY 11794

ROSENFELD, MARGARET, MD
PROFESSOR
DEPARTMENT OF PEDIATRICS
DIVISION OF PULMONARY MEDICINE
UNIVERSITY OF WASHINGTON SCHOOL OF MEDICINE
SEATTLE CHILDREN'S HOSPITAL
SEATTLE, WA 98105

SCHNEIDER, HARTMUT, MD, PHD *
ASSISTANT PROFESSOR
PULMONARY AND CRITICAL CARE
JOHNS HOPKINS UNIVERSITY
BALTIMORE, MD 21224

SMYTH, SUSAN S, MD, PHD *
PROFESSOR
DIVISION OF CARDIOVASCULAR MEDICINE
UNIVERSITY OF KENTUCKY
LEXINGTON, KY 40536

STEINER, MARIE ELIZABETH, MD
PROFESSOR
PEDIATRIC CRITICAL CARE
UNIVERSITY OF MINNESOTA
MINNEAPOLIS, MN 55455

WALL, DONNA A, MD *
PROFESSOR AND DIRECTOR
DEPARTMENT OF IMMUNOLOGY AND PEDIATRICS
UNIVERSITY OF MANITOBA
WINNIPEG, MB R3E 0V9
CANADA

SCIENTIFIC REVIEW OFFICER

COPE, KEARY A, PHD
SCIENTIFIC REVIEW OFFICER
OFFICE OF SCIENTIFIC REVIEW/DERA
NATIONAL HEART, LUNG, AND BLOOD INSTITUTE
BETHESDA, MD 20892

EXTRAMURAL SUPPORT ASSISTANT

HENDRICK, PATRICIA
PROGRAM SPECIALIST
OFFICE OF SCIENTIFIC REVIEW
NATIONAL HEART, LUNG, AND BLOOD INSTITUTE
BETHESDA, MD 20892

PROGRAM REPRESENTATIVE

BLAISDELL, CAROL J, MD
PROGRAM DIRECTOR
DIVISION OF LUNG DISEASES
NATIONAL HEART, LUNG, AND BLOOD INSTITUTE
BETHESDA, MD 20892

COOPER, LAWTON S., MD
HEALTH SCIENTIST ADMINISTRATOR
DIVISION OF CARDIOVASCULAR SCIENCES
NATIONAL HEART, LUNG, AND BLOOD INSTITUTE
BETHESDA, MD 29892

CURE, PABLO, MD, MPH
PROGRAM OFFICER
DIVISION OF BLOOD DISEASES & RESOURCES
NATIONAL HEART, LUNG, AND BLOOD INSTITUTE
BETHESDA, MD 20892

DESVIGNE-NICKENS, PATRICE, MD, PHD
MEDICAL OFFICIER
DIVISION OF CARDIOVASCULAR SCIENCES
NATIONAL HEART, LUNG, AND BLOOD INSTITUTE
BETHESDA, MD 20892

DI FRONZO, NANCY L, PHD
PROGRAM DIRECTOR
DIVISION OF BLODD DISEASES & RESOURCES
NATIONAL HEART, LUNG AND BLOOD INSTITUTE
BETHESDA, MD 20892

GHOFRANI, PEYVAND, PHD
CLINICAL TRIALS SPECIALIST
DIVISION OF LUNG DISEASES
NATIONAL HEART, LUNG, AND BLOOD INSTITUTE
BETHESDA, MD 20892

KAUFMANN, PETER G., PHD
HEALTH SCIENCE ADMINISTRATOR
DIVISION OF CARDIOVASCULAR SCIENCES
NATIONAL HEART, LUNG, AND BLOOD INSITITUTE
BETHESDA, MD 20817

NICASTRO, HOLLY L., PHD
HEALTH SCIENTIST ADMINISTRATOR
DIVISION OF CARDIOVASCULAR SCIENCES
NATIONAL HEART, LUNG, AND BLOOD INSTITUTE
BETHESDA, MD 20892

REINECK, LORA A., MD
MEDICAL OFFICER
DIVISION OF LUNG DISEASES
NATIONAL HEART, LUNG, AND BLOOD INSTITUTE
BETHESDA, MD 20892

SOPKO, GEORGE, MD
MEDICAL OFFICER
DIVISION OF CARDIOVASCULAR SCIENCES
NATIONAL HEART, LUNG, AND BLOOD INSTITUTE
BETHESDA, MD 20892

WERNER, ELLEN M, PHD
PROGRAM DIRECTOR
DIVISION OF BLOOD DISEASES AND RESOURCES
NATIONAL HEART, LUNG, AND BLOOD INSTITUTE
BETHESDA, MD 20892

GRANTS MANAGEMENT REPRESENTATIVE

JESSEE, DIANNA
GRANTS MANAGEMENT SPECIALIST
OFFICE OF GRANTS MANAGEMENT
NATIONAL HEART, LUNG, AND BLOOD INSTITUTE
BETHESDA, MD 20892

OBSERVER

TINSLEY, EMILY
DIVISION OF CARDIOVASCULAR SCIENCES
NATIONAL HEART, LUNG, AND BLOOD INSTITUTE
BETHESDA, MD 20892

* Temporary Member. For grant applications, temporary members may participate in the entire meeting or may review only selected applications as needed.

Consultants are required to absent themselves from the room during the review of any application if their presence would constitute or appear to constitute a conflict of interest.
